# Polymerization-Inhibited Twisted Intramolecular Charge Transfer for Strong Molecular Aggregate Emission

**DOI:** 10.1021/acspolymersau.5c00123

**Published:** 2025-11-05

**Authors:** Suiying Ye, Carolina Söll, Wanqing Cao, Benjamin Choiselat, Ramsha Khan, Tero-Petri Ruoko, Yinyin Bao

**Affiliations:** † Department of Chemistry and Applied Biosciences, 27219 ETH Zurich, Vladimir-Prelog-Weg 1-5/10, 8093 Zurich, Switzerland; ‡ Department of Chemistry, Faculty of Science, 105417 University of Helsinki, A. I. Virtasen Aukio 1, 00014 Helsinki, Finland; § Chemistry and Advanced Materials, Faculty of Engineering and Natural Sciences, 4566 Tampere University, 33720 Tampere, Finland

**Keywords:** twisted intramolecular charge transfer, light-emitting polymers, aggregation-induced emission, solid-state emission, controlled radical polymerization, molecular aggregates

## Abstract

Molecular fluorophores exhibiting intramolecular charge transfer (ICT) processes are of great interest across diverse fields, as engineering electron donor or acceptor groups enables control over fluorescence intensity and emission color. However, the fluorescence quantum yield is often decreased by the formation of twisted intramolecular charge transfer (TICT) states. Conventional strategies for reducing TICT effects typically require toxic, expensive reagents and complex syntheses. Here, we present a simple and robust approach that leverages electron-rich, rigid polymer chains to suppress the TICT of naphthalimide-based fluorophores, achieving strong molecular emission in aggregate and solid states. The polymerization-inhibited TICT strategy imparts strong aggregation-induced emission (AIE) behavior in the resulting oligomers and polymers, regardless of the inherent AIE activity of the fluorophore, yielding solid-state photoluminescence quantum yields of up to 0.80. Moreover, the TICT inhibition upon aggregation was indicated simultaneously by the significant blue shift of the emission wavelength as well as by the increased fluorescence lifetime. This work establishes a cost-efficient and versatile methodology for developing highly emissive materials with potential in optoelectronics, such as luminescent solar concentrators and waveguides. We anticipate expanding this approach to a wider range of TICT dyes beyond naphthalimide derivatives.

## Graphical abstract

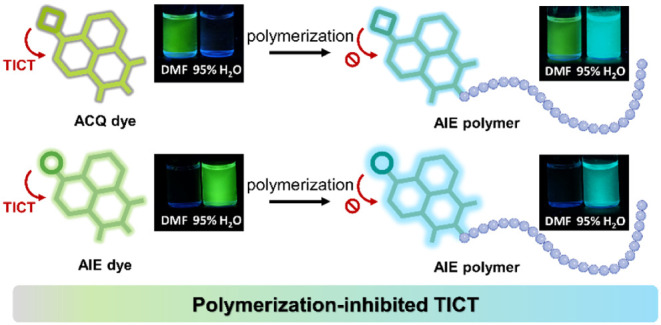

## Introduction

Organic fluorescent molecules and polymers have attracted significant interest in recent decades. Their remarkable versatility stems from the extensive range of molecular designs and material processing options.^1,2^ Exhibiting immense potential across a spectrum of applications, these materials have established their importance in various fields such as chemical sensing,^3^ bioimaging,^4^ OLEDs,^5,6^ and light-harvesting technologies.^7^ Therefore, the precise control of the fluorescence intensity and emission color of these molecular fluorophores, particularly in solid or aggregated states, holds significant value for both academic research and practical applications. To achieve this, numerous molecular fluorophores are designed with push–pull (electron-donating and -withdrawing) functional groups located at opposite ends of a π-conjugated aromatic core. This arrangement fosters intramolecular charge-transfer (ICT) properties in the excited state, allowing for the manipulation of fluorescence properties by adjusting the electron donor and acceptor groups.^8–10^ However, the formation of the twisted ICT (TICT) excited state in ICT systems can weaken their fluorescence.^11–14^

To address this challenge, extensive efforts have been made in electron donor engineering to reduce the TICT effect and thereby enhance the photoluminescence quantum yield (Φ). For instance, cyclizing the alkylamino donor groups and thereby increasing the rigidity of coumarin derivatives can significantly suppress the formation of TICT states, leading to an improved Φ ranging from 0.66 to 1.0 in organic solvents and water.^15^ Additionally, incorporating electron-donating groups with a small ring size, such as an azetidinyl group, has demonstrated a significant enhancement in the fluorescence of rhodamine, coumarin, and naphthalimide (NPT) derivatives in aqueous solutions, as reported by Lavis and coworkers.^16^ This enhancement is attributed to the minimized steric clash between the donor group and the fluorophore scaffold; which, otherwise, could allow intramolecular rotations that promote the emergence of the detrimental TICT state. While these strategies have demonstrated success, they often necessitate highly toxic or expensive reagents (e.g., aziridine)^13^ or multistep organic syntheses (e.g., cyclization of alkylamino groups),^12^ coming with increased costs and environmental concerns. On the other hand, while electron donor engineering is often utilized for fluorescence amplification in aqueous solutions, it exhibits limited effectiveness in achieving strong molecular emission in the solid-state.^14^

Concurrently, significant efforts have been dedicated to developing ICT molecules that exhibit aggregation-induced emission (AIE)^17^ activity. The incorporation of AIE-active moieties in ICT molecules can notably amplify their fluorescence in aqueous aggregates or solid states.^18,19^ This is likely due to the suppression of the TICT effect enabled by the aggregation process. As a result, these molecules exhibit TICT properties in organic solvents and AIE characteristics in solid or aggregate states. For example, Zhu and coworkers reported that by conjugating one or two tetraphenylethene units with the NPT scaffold, two ICT derivatives were obtained, exhibiting solid-state quantum yields of 0.76 and 0.57, respectively.^20^ Nevertheless, incorporating the AIE effect does not always guarantee enhanced solid-state emission. Designing ICT molecules with solid-state quantum yields exceeding 0.70 using AIE-based approaches remains generally challenging.^18^

We previously discovered that growing short polymer chains with varied electron densities from electron-accepting fluorophores can significantly influence the charge transfer processes within the polymer systems, thereby enabling precise control of their aggregate emission properties.^21–23^ Building on this, we hypothesized that introducing electron-rich, rigid polymer chains into TICT molecules could potentially modulate the charge transfer processes, and if the TICT degree can be effectively restricted, enhanced aggregate emission may be realized. To test this hypothesis, we selected two naphthalimide (NPT)-based TICT dyes as model fluorophores and grew polystyrene of varied chain length onto them via atom transfer radical polymerization (ATRP). Interestingly, the polymerization process endowed substantial AIE properties to these ICT molecules, regardless of their inherent AIE activity or lack thereof (Figure 1). As a result, the polymer solid films exhibited solid-state quantum yields as high as 0.80.

**Figure 1:**
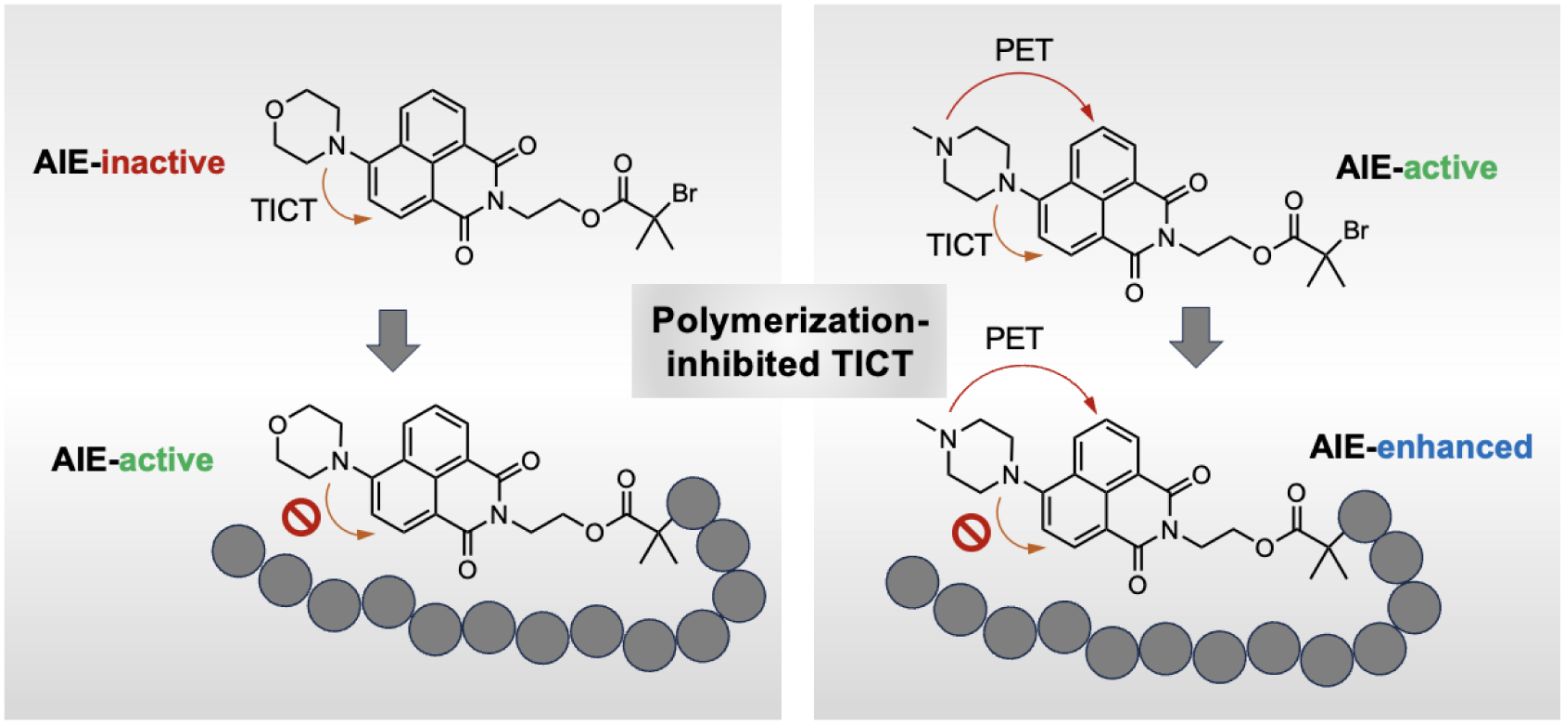
Schematic illustration of polymerization-inhibited TICT enabling the transitions of an AIE-inactive NPT into AIE-active polymers and an AIE-active NPT into AIE-enhanced polymers, respectively.

Our approach involves only low-cost, commercially available reagents and extremely easy synthesis. It is significantly different from the conventional methods relying on physically embedding dye molecules in polymer matrice,^24,25^ or systems with large dilution of dye molecules.^26^ For instance, the Φ_film_ of NPT oligomer reached 0.71 even at a high dye concentration of nearly 20 wt % (degree of polymerization = 20 and molecular weight = 2550 g·mol^–1^). Furthermore, a notable blue shift in emission wavelength (from 540 to 500 nm) and increased fluorescence lifetime were also observed that are signature behaviors of systems incorporating both AIE and TICT.^27^ We believe that the incorporated short polymer chains can efficiently inhibit the TICT process upon aggregation, leading to significantly enhanced solid-state emission.

## Experimental Section

### Materials

Unless stated otherwise, all reagents were used as received without further purification. *N*,*N*-Dimethylformamide (DMF, 99.8% extra dry over molecular sieves), xylenes (mixture of isotopes), dichloromethane (DCM), styrene (St), and dimethyl sulfoxide (DMSO), 2-methoxyethanol (99+% extra dry) were purchased from Acros Organics (Fair Lawn, NJ, USA). Copper­(I) bromide (CuBr) was obtained from ABCR (Karlsruhe, Deutschland). 4,4’-Dionyl-2,2’-dipyridyl (dNbpy), 1-methylpiperazine (Mpp), and 1-vinyl-naphthalene (Naph) were obtained from TCI (Tokyo, Japan). Methanol (MeOH), mesitylene, benzene, aluminum oxide basic, 2-aminoethanol, α-bromoisobutyryl bromide, hexane, tetrahydrofuran (THF), morpholine (Mph), and silica gel (for column chromatography) were purchased from Aldrich (St. Louis, MO, USA). Ethanol (EtOH) and *N*,*N*-dimethylformamide (DMF) were obtained from Supelco (Bellefonte, PA, USA). Triethylamine (Et_3_N) and methoxyethanol were purchased from Thermo Scientific (Waltham, MA, USA). 4-Brominated naphthalic anhydride was obtained from Apollo Scientific (Whitefield, UK). Acetone-d6 (99.9%, deuterated), chloroform-d (CDCl_3_, 99.8%, deuterated), and dimethyl sulfoxide-d6 (DMSO-d6, 99.9%, deuterated) were purchased from Cambridge Isotope Laboratories Inc. (Tewksbury, MA, USA).

### Synthesis

#### Monomers

Summaries of the naphthalimide-based atom transfer radical polymerization (ATRP) initiator syntheses are provided in Scheme 1 for MphNPT and Scheme 2 for MppNPT. To obtain 6-bromo-2-(2-hydroxyethyl)­benzo­[de]­isoquinoline-1,3-dione (BrNPT–OH), the following steps were taken. First, 4-brominated naphthalic anhydride (5.5 g, 20 mmol) and 1-aminoethanol (6 mL, 100 mmol) were dissolved in 150 mL of ethanol. The reaction mixture was then stirred overnight at 80 °C, followed by pouring into cold deionized water. The resulting precipitate was washed with 50 mL of ethanol, yielding a light-yellow solid with 83% yield. The product was characterized by ^1^H NMR (400 MHz, DMSO) δ 8.61–8.52 (m, 2H), 8.35 (d, J = 7.9 Hz, 1H), 8.23 (d, J = 7.9 Hz, 1H), 8.01 (dd, J = 8.5, 7.3 Hz, 1H), 4.81 (t, J = 6.0 Hz, 1H), 4.15 (t, J = 6.5 Hz, 2H), 3.63 (q, J = 6.3 Hz, 2H).

**Scheme 1:**
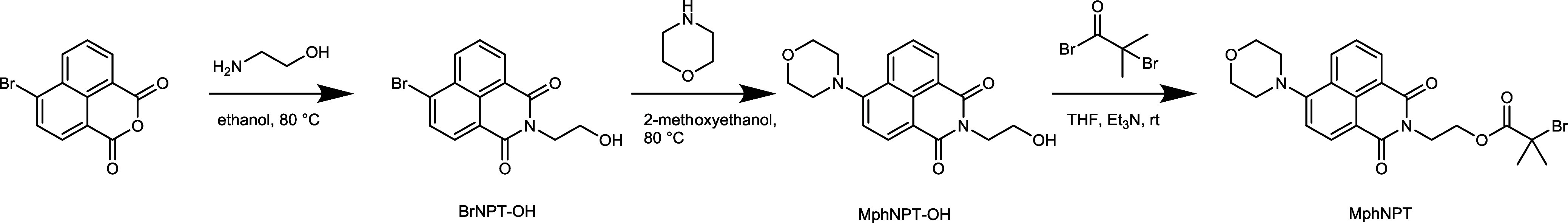
Synthetic Route of MphNPT

BrNPT–OH (2.5 g, 7.8 mmol, 1 equiv) was dissolved in 50 mL of 2-methoxyethanol. Either morpholine (13.5 mL, 156.5 mmol, 20 equiv) or 1-methylpiperazine (8.5 mL, 76.6 mmol, 10 equiv) was then added to the solution. The mixture was stirred overnight at 130 °C. Following this, the solution was concentrated using a rotary evaporator at 50 °C and 50–60 mbar. After concentration, 10 mL of acetone was added, and crystals formed in the freezer over the course of several days. The crystals were subsequently collected and washed with ethanol. Both products were obtained as yellow crystals. MphNPT–OH was recovered with a yield of 87%, while MppNPT–OH was obtained with a yield of 42%. MphNPT–OH: ^1^H NMR (400 MHz, CDCl_3_) δ 8.61 (dd, J = 7.3, 1.2 Hz, 1H), 8.55 (d, J = 8.1 Hz, 1H), 8.44 (dd, J = 8.5, 1.2 Hz, 1H), 7.72 (dd, J = 8.4, 7.3 Hz, 1H), 7.24 (d, J = 8.1 Hz, 1H), 4.46 (dd, J = 5.7, 4.8 Hz, 2H), 4.06–3.94 (m, 7H), 3.32–3.21 (m, 5H), 2.48 (s, 1H). MppNPT–OH: ^1^H NMR (400 MHz, CDCl_3_) δ 8.60 (dd, J = 7.3, 1.2 Hz, 1H), 8.53 (d, J = 8.1 Hz, 1H), 8.43 (dd, J = 8.5, 1.2 Hz, 1H), 7.70 (dd, J = 8.4, 7.3 Hz, 1H), 7.23 (d, J = 8.1 Hz, 1H), 4.46 (dd, J = 5.7, 4.7 Hz, 2H), 3.97 (q, J = 5.4 Hz, 2H), 3.32 (t, J = 4.8 Hz, 4H), 2.75 (s, 4H), 2.52 (t, J = 5.5 Hz, 1H), 2.44 (s, 3H).

The introduction of the α-bromoisobutyryl group was performed as follows: MphNPT–OH (1.9 g, 5.8 mmol, 1 equiv) and Et_3_N (0.974 mL, 7.0 mmol, 1.2 equiv) were dissolved in 60 mL of THF. A solution of α-bromoisobutyryl bromide (0.9 mL, 7.0 mmol, 1.2 equiv) in 2 mL of THF was added slowly to the mixture. After stirring at room temperature (rt) overnight, the mixture was poured into cold deionized water. The workup process involved first extractions using DCM and washing with NaHCO_3_ and NaCl solutions. The collected organic phase was dried over MgSO_4_ and then concentrated using a rotary evaporator. Column chromatography on silica gel was performed with a solvent mixture of MeOH/DCM (1/100, v/v). The product was further purified by recrystallization from ethanol. The final product, MphNPT, was obtained as a yellow solid with a yield of 70%. ^1^H NMR and ^13^C NMR spectra (400 MHz, CDCl_3_) were displayed in Figures S14 and S15. For MppNPT, MppNPT–OH (1.5 g, 4.4 mmol, 1 equiv) and Et_3_N (0.74 mL, 5.3 mmol, 1.2 equiv) were dissolved in 40 mL of THF. A solution of α-bromoisobutyryl bromide (0.7 mL, 5.3 mmol, 1.2 equiv) in 2 mL THF was added slowly to the mixture. After stirring at room temperature overnight, the mixture was poured into cold deionized water and then extracted six times with DCM, including washing with NaHCO_3_ and NaCl solutions. The organic phase was dried with MgSO_4_ and concentrated on the rotary evaporator. Column chromatography on silica gel was performed with MeOH/DCM (1/10, v/v). The product MppNPT was obtained as a yellow solid with a yield of 82%. ^1^H NMR and ^13^C NMR spectra (400 MHz, CDCl_3_) were displayed in Figures S19 and S20 with detailed attributions. High-resolution mass spectrometry (ESI+): MphNPT, *m*/*z* (mass/charge ratio) = 475.08529 (M+H)^+^, 497.08025 (M+Na)^+^; MppNPT, *m*/*z* (mass/charge ratio) = 488.11607 (M+H)^+^;

#### Polymers

For the polymer syntheses, MphNPT or MppNPT were utilized as ATRP initiators. The polymer chain length was controlled by varying the monomer-to-initiator ratios or by adjusting the reaction time. The detailed experimental conditions and polymerization controls are reported in Table S1. In a typical polymerization procedure, the initiator (1 equiv) was placed in a dry Schlenk flask. Copper­(I) bromide (1 equiv) and dNbpy (1 equiv) were added, and the flask was evacuated for 30 min. Several vacuum and argon backfill cycles were then performed to remove oxygen. Meanwhile, styrene was deoxygenated by bubbling argon for 10 min. For copolymerization, styrene and 1-vinylnaphthalene were weighed into a small flask and bubbled with argon. The desired amount of monomer and/or comonomer were then added to the Schlenk flask under argon using a syringe, followed by heating at 80 or 115 °C in an oil bath. After stirring for a specific time, aliquots were taken from the flask and cooled to stop the polymerization. The resulting brown reaction mixtures were dissolved in THF and filtered through an alumina column. The THF was then evaporated using a rotary evaporator, and the concentrated solution was precipitated once with hexane and twice with methanol. The powders were collected and dried under vacuum. The purified polymers were characterized using ^1^H NMR and size exclusion chromatography (SEC), the spectra are shown in Figures S16–S18 for MphNPT polymers and Figures S21–S23 for MppNPT polymers, and the number-average molecular weight (*M*_n_) and dispersity index (*Đ*) are listed in Table S1. We note a good consistency between the *M*_n_ reported by NMR and SEC (Table S1), and the polymers showed low polydispersity index, with *Đ* values around 1.1 (Figures S19 and S24). These indicate good polymerization controls of the dye-initiated ATRP method.

### Characterizations

Proton Nuclear Magnetic Resonance (^1^H NMR) spectra were recorded on an AV-400 400 MHz spectrometer (Bruker, Billerica, MA, USA) at room temperature. ^13^C NMR spectra were recorded on a Bruker Ultrashield Plus 500 MHz spectrometer (125 MHz). Electrospray ionization mass spectrometry (ESI-MS) experiments were conducted using an Agilent 6230 time-of-flight liquid chromatography/MS system using positive mode. One μg/mL Methanol/Milli-Q (50/50, v/v) was used as the mobile phase at a flow rate of 5 μL/min. The capillary voltage was set to 4.0 kV. The capillary gap temperature was held at 325 °C. An Agilent MassHunter workstation was used for data acquisition and processing.

Size exclusion chromatography (SEC) measurements were carried out using Waters Acquity APC system. The system consisted of Acquity Column Manager: S, Sample Manager: pFTN, p: Isocratic Solvent Manager, Acquity RI Detector and Acquity TUV Detector. Column used was Agilent Polargel L (7.6 × 300 mm) and the flow rate was 0.800 mL/min, eluent DMF + LiBr 1g/l. DMF was HPLC grade supplied by Fischer Scientific and PMMA standards were supplied by Polymer Standards Service. Empower 3 software was used to analyze the results.

UV–vis absorption spectra were recorded with JASCO V-750 spectrophotometer. One mM stock solutions in DMF were prepared for all of the samples. They were diluted into 10 μM DMF or 10 μM DMF/H_2_O = 5/95 solutions for the UV–vis test. The bandwidth of 1.0 nm, response duration of 0.24 s, and scan speed of 158 nm/min were used. At 350 nm, the light source was changed from a deuterium lamp to a halogen lamp. Photoluminescence (PL) spectra of samples in solutions/suspensions were obtained with a Cary Eclipse fluorescence spectrophotometer or JASCO FP-8550 spectrofluorometer for D_2_O-based samples. One mM stock solutions in DMF were prepared for all of the samples. They were diluted into 10 μM DMF or 10 μM DMF/D_2_O = 5/95 solutions for the photoluminescence test. An excitation wavelength of 380 nm, scan speed of 500 nm/min, excitation bandwidth of 5 nm, and emission bandwidth of 5 nm were used. The tests were conducted at 25 °C. Blank correction was performed before the test to eliminate the influence of the solvents. SpectraManager was used for data acquisition and processing.

Time-resolved photoluminescence (TRPL) was measured using the time-correlated single photon counting method with a PicoQuant FluoTime 300 spectrometer equipped with a Peltier-cooled photomultiplier tube for detection and excited using an LDH-D-C-405 laser diode (emission at 401 nm with sub-50 ps pulse fwhm) driven with the PDL 820 laser driver. PL spectra of thin film samples were characterized using a Hamamatsu Quantaurus-Tau Fluorescence Lifetime Spectrometer (C11367–31) equipped with a photon counting measurement system. The absolute photoluminescence quantum yields (PLQYs) of spin-casted thin films were determined using the Quantaurus QY (C11347-11) from Hamamatsu equipped with a 150 W xenon light source and a 3.3-in. (8.38 cm) integrating sphere, which is coated with highly reflective Spectralon. For thin films produced with polymers, a typical spin-casting method was applied using a polymer solution in toluene (20 mg/mL). Specifically, quartz substrates with a size of 10 × 10 mm were brush-cleaned using the mixture of Extran MA02 soap solution and water (1:3, v/v). Subsequently, all substrates were sonicated in acetone and isopropanol, respectively. These substrates were then dried on a hot plate at 120 °C for 20 min. Finally, the sample solutions were spin-cast at a spin rate of 1500 rpm for 40 s.

## Results and Discussion

NPT derivatives are a representative group of TICT dyes with a highly tunable chemical structure and photophysical properties,^28,29^ which have been widely investigated for sensing, imaging, and optoelectronics.^30–32^ We first selected the naphthalimide derivative functionalized with a morpholine moiety at the 4-position of the naphthalimide core, which is a typical TICT fluorescent dye and has been employed in cell imaging.^33–36^ An α-bromoisobutyryl substituent at the imide nitrogen was further introduced, resulting in an ATRP initiator named MphNPT, to enable subsequent polystyrene growth, yielding MphNPT-PS­(*n*), where *n* is the obtained degree of polymerization (DP) (Figure 2A and Scheme 1).

**Figure 2:**
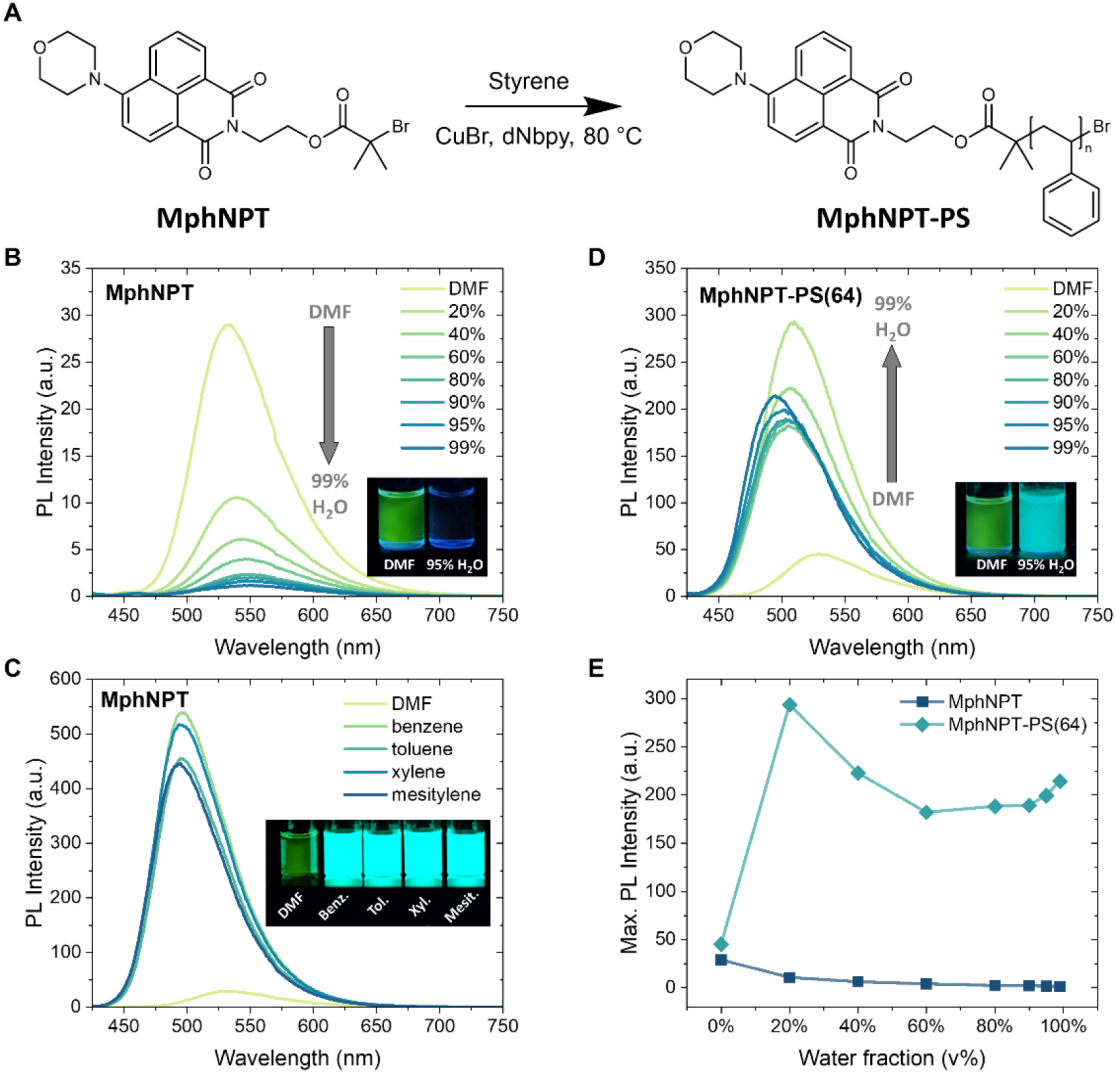
Photophysical properties of MphNPT and MphNPT-PS. (A) Chemical structure of the initiator MphNPT and the polymer MphNPT-PS via ATRP. (B) PL spectra of MphNPT in DMF and DMF/water mixtures with different water fractions. Inset: Photograph of MphNPT in DMF and in DMF/water with 95% water fraction under UV light (365 nm). (C) PL spectra of MphNPT in DMF and different aromatic solvents. Inset: Photograph of MphNPT in DMF and different aromatic solvents under UV light (365 nm). (D) PL spectra of MphNPT-PS(64) in DMF and DMF/water mixtures with different water fractions. Inset: Photograph of MphNPT-PS(64) in DMF and in DMF/water with 95% water fraction under UV light (365 nm). (E) Comparison of the maximum PL intensity of MphNPT and MphNPT-PS(64) in DMF or DMF/water mixtures with different water fractions. The concentration was 5.0 μM, and the excitation wavelength was λ_ex_ = 400 nm.

Unlike small aziridine rings^13^ or bulky groups,^28,37^ the morpholine unit was found to provide limited resistance against TICT rotations, resulting in a low emission quantum yield in polar solvents (∼0.05).^13^ Consistent with previous observations, MphNPT exhibits decreased emission intensity and red-shifted emission wavelength when increasing solvent polarity (Figure S1), attributed to solvent relaxation effects. To further probe the aggregation effects, we systematically increased the water fraction from 0 to 99% (v/v) in DMF/water mixtures. The fluorescence intensity gradually decreased with rising water content, indicative of a typical aggregation-caused quenching (ACQ) process (Figure 2B). A control experiment comparing the emission of MphNPT in DMF and in DMF/D_2_O (5/95, v/v) showed similar quenching behavior (Figure S2), reinforcing the interpretation of ACQ rather than solvent-assisted quenching due to energy transfer or hydrogen bonding.^38^ The quenching of fluorescence is attributed to the strong TICT effect and also likely to the formation of H-aggregates^28^ of MphNPT in poor solvents, as indicated by the red-shifted absorption maxima and the broadened absorption spectrum upon aggregation (Figure S3). On the other hand, enhanced blue-shifted fluorescence was observed in aromatic solvents (Figure 2C) due to their lower polarity that reduces the degree of TICT. In aromatic solvents of differing electron-donating ability, the emission wavelengths at maximum intensity are similar to each other, indicating negligible charge transfer between the solvent molecules and MphNPT.

Next, we investigated the potential role of polymer chains in tuning the photoluminescence of MphNPT (Figure 2A). The aggregate emission of MphNPT-PS(64) was first studied in DMF/water mixtures (Figure 2D). Drastically different from the small molecule MphNPT, the polymer displayed pronounced AIE behavior rather than the ACQ effect. The photoluminescence (PL) intensity increased upon water addition, reaching a maximum at 20% water fraction with a ∼6-fold enhancement relative to the DMF solution and ∼30-fold higher than the small molecule MphNPT under the same conditions. Upon further increasing the water fraction up to 60%, a slight decrease in emission intensity was found, likely due to polarity-induced stabilization of the ICT state. From 60% on, fluorescence intensity increased again. We hypothesize that under these highly aggregated conditions, the polymer chains provide steric hindrance to prevent the formation of H-aggregates and simultaneously provide a nonpolar microenvironment that reduces the degree of TICT of the fluorophore, thereby maintaining high fluorescence intensity in the aggregate state (Figure 2E). A control experiment in DMF and DMF/D_2_O (5/95, v/v) was also performed, showing similar AIE activity of MphNPT-PS(64) (Figure S2).

In contrast to a red-shifted absorption maximum in MphNPT, the absorption spectra of MphNPT-PS showed a slight blue shift in the aggregate state compared to its dissolved state, despite the scattering peaks from aggregated particles (Figure S3). This implies trivial alternation on molecular ground-state interactions after polymerization. To probe the excited-state processes, we performed time-resolved photoluminescence (TRPL) measurements. The TRPL results of MphNPT in DMF (Figure 3A, see Table S3 for fitting details) illustrate fast radiative recombination with a lifetime of **τ**_**1**_ = 0.88 ns along a minor (relative amplitude of 1.3%) longer living component with a lifetime of **τ**_**2**_ = 7.7 ns (**τ**_**avg**_ = 1.57 ns). The fast emission component can be assigned to locally excited (LE) emission, whereas the very weak, longer living component presumably originates from the TICT state. Increasing the water fraction dramatically decreases the LE emission lifetime (Figure 3B), consistent with increased polarity of the solvent mixture stabilizing ICT. Only the very weak emission from the TICT state is visible for solutions with a water fraction ≥ 40%. On the other hand, the polymer MphNPT-PS(18) in DMF (Figure 3C) showed a better fit with a three-exponential model, obtaining **τ**_**1**_ = 0.64, **τ**_**2**_ = 1.60, and **τ**_**3**_ = 7.65 ns (**τ**_**avg**_ = 1.81 ns). The **τ**_**1**_ component has approximately half the amplitude of **τ**_**2**_, and we assign these two components to a seemingly biexponential LE emission, whereas the minor (relative amplitude of 1.1%) long-living **τ**_**3**_ component is again assigned to emission from the TICT state. When the water fraction is increased, the lifetime and relative amplitude of the fast component remain largely the same, whereas the lifetimes of the two slower components increase to **τ**_**2**_ = 4.54 and **τ**_**3**_ = 9.32 ns already at 20% water fraction. This is indicative that the fitting now preferentially fits the AIE with two exponential components. For this reason, we plotted the intensity-weighted average lifetimes of all three components at different water fractions in Figure 3D. The average lifetime quickly saturates to **τ**_**avg**_ ≈ 7.7 ns with increased water fraction, indicative of slower AIE emission dominating the emission profile of MphNPT-PS(18) at the aggregate state. Altogether, by initiating polymerization of styrene from a morpholine-functionalized naphthalimide, we successfully converted an intrinsically ACQ-type fluorophore into a robust AIE-active macromolecular system, achieving over an order-of-magnitude increase in emission intensity in aggregated states.

**Figure 3:**
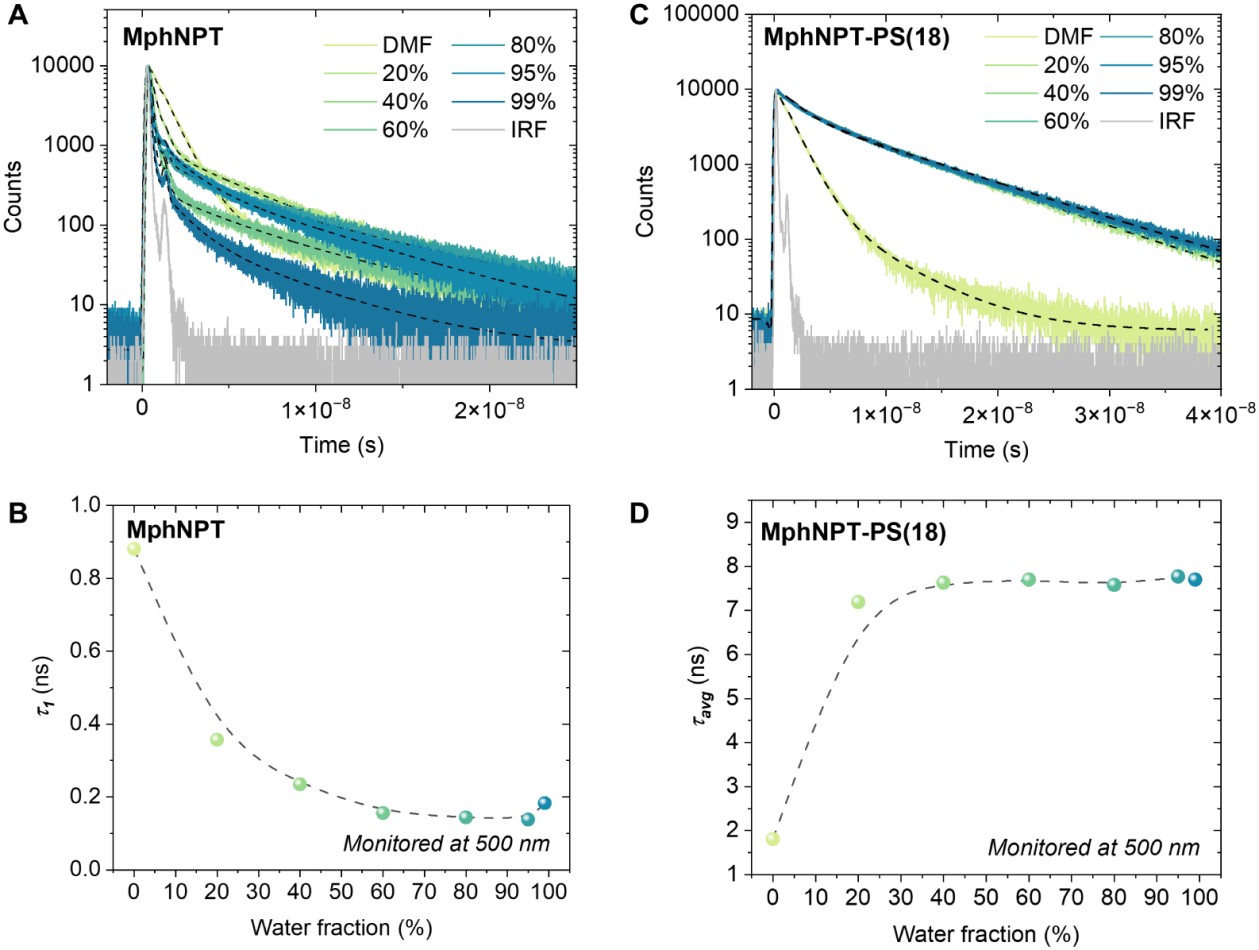
TRPL measurements of MphNPT and MphNPT-PS in DMF and DMF/water mixtures with different water fractions. (A) TRPL decays of MphNPT in DMF and DMF/water mixtures with different water fractions. (B) Magnified TRPL decays of MphNPT in DMF and DMF/water (5/95, v/v) (C) TRPL decays of MphNPT-PS(18) in DMF and DMF/water mixtures with different water fractions. (D) Intensity weighted average lifetimes of MphNPT-PS(18) emission with different water fractions. The concentration was 15.0 μM, the excitation wavelength was λ_ex_ = 401 nm, and the emission was monitored at 500 nm.

To further reveal the role of the polymer chains in restoring the high fluorescence intensity via steric hindrance, we investigated the correlation between the photophysical properties and the polymer chain length (DP = 12, 18, 20, and 64). All polymers showed strong fluorescence in the aggregate state (Figure S4) with negligible absorption shifts (Figure S5), in line with previous observations. It was found that the fluorescence intensity of the polymers increased with chain growth, accompanied by a blue shift in emission maxima (Figure S4).

We further measured the solid-state emission spectra and PL quantum yield (PLQY, Φ) of polymer films by spin-coating from toluene solutions (Figure 4). While the Φ of the initiator is 0.44 in film, the oligomer with DP 12 showed a Φ value of 0.51, followed by increasing Φ values of 0.71 and 0.80 with polymers of DP 20 and 64, respectively (Figure 4B). Additionally, clear blue shifts were also observed when increasing the DP from 0 (i.e., the initiator) to 64, with a maximum emission wavelength continuously decreasing from 533 to 497 nm (Figure 4B). This can be clearly seen from the yellow to blue color changes of the initiator/polymer powders (Figure 4A inset). These results highlight that the nonpolar polymer chains with steric hindrance distort the stacking of the fluorophore, and thus inhibit TICT, leading to enhanced PL emission in both aggregate and solid state. Note that further increase in DP beyond the present systems should be less effective for emission enhancement, as indicated by the flatter increase of Φ values from DP 20 to 64 (Figure 4B). We emphasize that a relatively low DP (e.g., 20, dye wt % of nearly 20 wt %) was able to provide sufficient steric hindrance and thereby remarkably increase Φ values.

**Figure 4:**
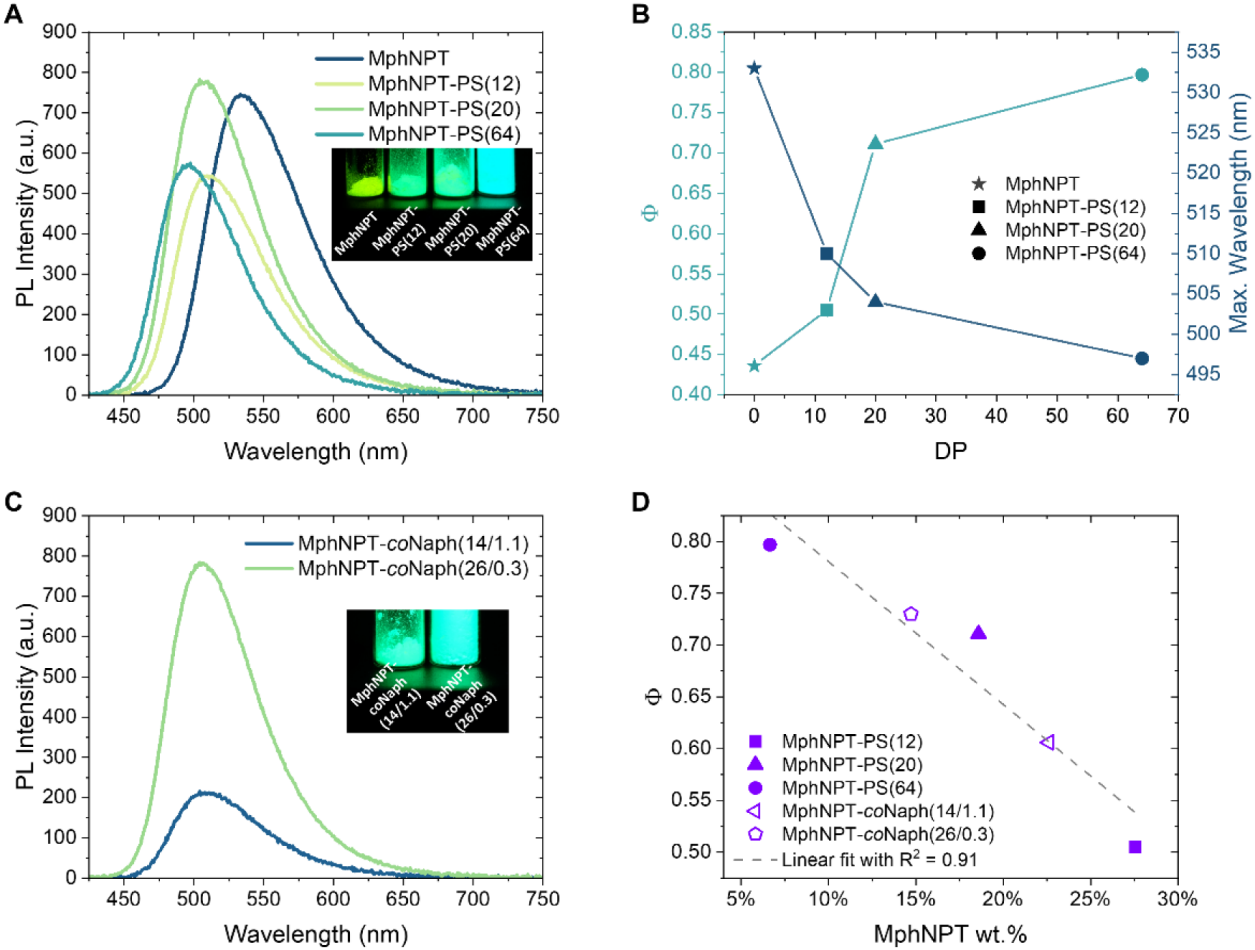
Photophysical properties of MphNPT-PS and MphNPT-*co*Naph of different DPs. (A) PL spectra of solid films produced with MphNPT and MphNPT-PS of different DPs. Inset: Photograph of solid powders of MphNPT and MphNPT-PS of different DPs under UV light (365 nm). (B) The solid-state Φ and the wavelength of maximum emission, plotted against DP (DP = 0 corresponds to MphNPT). (C) PL spectra of solid films produced with MphNPT-*co*Naph of different DPs. Inset: Photograph of solid powders of MphNPT-*co*Naph of different DPs under UV light (365 nm). (D) The Φ of polymer films, plotted against weight fraction (wt %) of MphNPT. The excitation wavelength was λ_ex_ = 400 nm.

On the other hand, monomer effects were investigated by preparing copolymers of styrene and 1-vinylnaphthalene, denoted as MphNPT-*co*Naph­(*x*/*y*), where *x*, *y* is the calculated average DP of styrene and 1-vinylnaphthalene, respectively (Figure S6A). These copolymers again exhibited strong emission in aggregate (Figure S6B) and solid state (Figure 4C). When compared with MphNPT-PS of similar DP, nearly identical emission maxima were observed (Figure S6C). These results also suggested that the steric effects and the microenvironment of polymer chains—varied by DP—play a dominant role in emission enhancement, while charge transfer interactions between the acceptor type NPT fluorophore and strong electron donors (e.g., naphthalene) are negligible (Figure S6D,E). This also confirmed that it is possible to introduce functional monomers into this polymer system without interrupting the emission behavior.

To further validate this interpretation, the MphNPT fluorophore weight fraction (wt %) in polymers/copolymers was quantified and correlated with the thin film Φ (Figure 4D). A clear linear trend (*R* ^2^ = 0.91) was observed, with Φ rising from 0.51 to 0.80 upon reducing the dye concentration. Further dilution was investigated by preparing thin films of mixtures of MphNPT-PS(64) and polystyrene or poly­(methyl methacrylate), with highest Φ up to 0.88 recorded at 50 wt % in PMMA (Figure S7). This demonstrates the potential of polymerization-inhibited TICT for achieving strong solid-state molecular emission.

With these promising results in mind, we next turned our interest to another NPT derivative functionalized with *N*-methylpiperazine, which has both TICT and photoinduced electron transfer (PET) processes occurring simultaneously within the fluorophore. Despite minimal differences in chemical structure from morpholine-functionalized NPT, *N*-methylpiperazine-substituted NPT often exhibits AIE activity,^39^ due to the inhibition of TICT when transitioning to the aggregate state.^39,40^ To verify if our polymerization strategy could be extended to fluorophores exhibiting both TICT and PET, we synthesized another ATRP initiator, MppNPT, bearing an α-bromoisobutyryl group at the imide position, and prepared the corresponding polymers (MppNPT-PS) (Figure 5A and Scheme 2). As expected, MppNPT showed typical AIE behavior with enhanced PL intensity upon increasing the water fraction in DMF/water mixtures (Figure 5B) as well as enhanced emission in nonpolar, aromatic solvents (Figure S8). Similar broadening and slight red shifts in absorption spectra in the aggregate states again reflected the cooperative roles of TICT and AIE upon molecular stacking (Figure S10). A control experiment in DMF and DMF/D_2_O mixtures confirmed that the AIE effect was not influenced by hydrogen bonding (Figure S9).

**Scheme 2:**
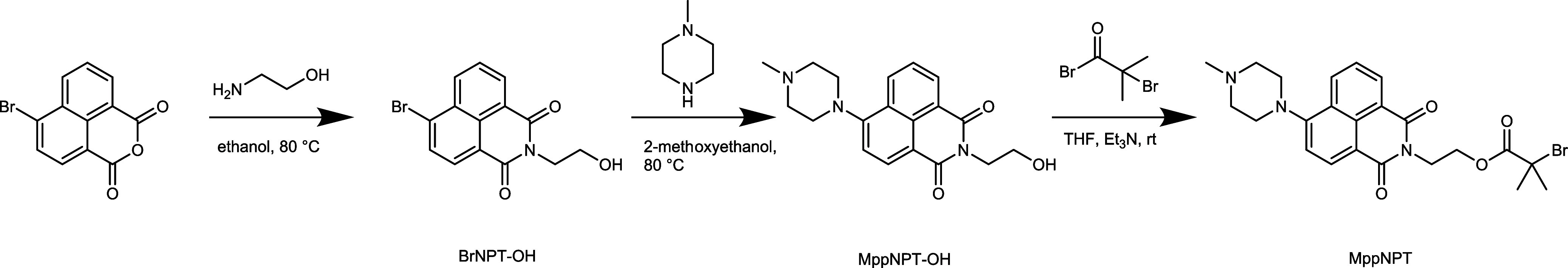
Synthetic Route of MppNPT

**Figure 5:**
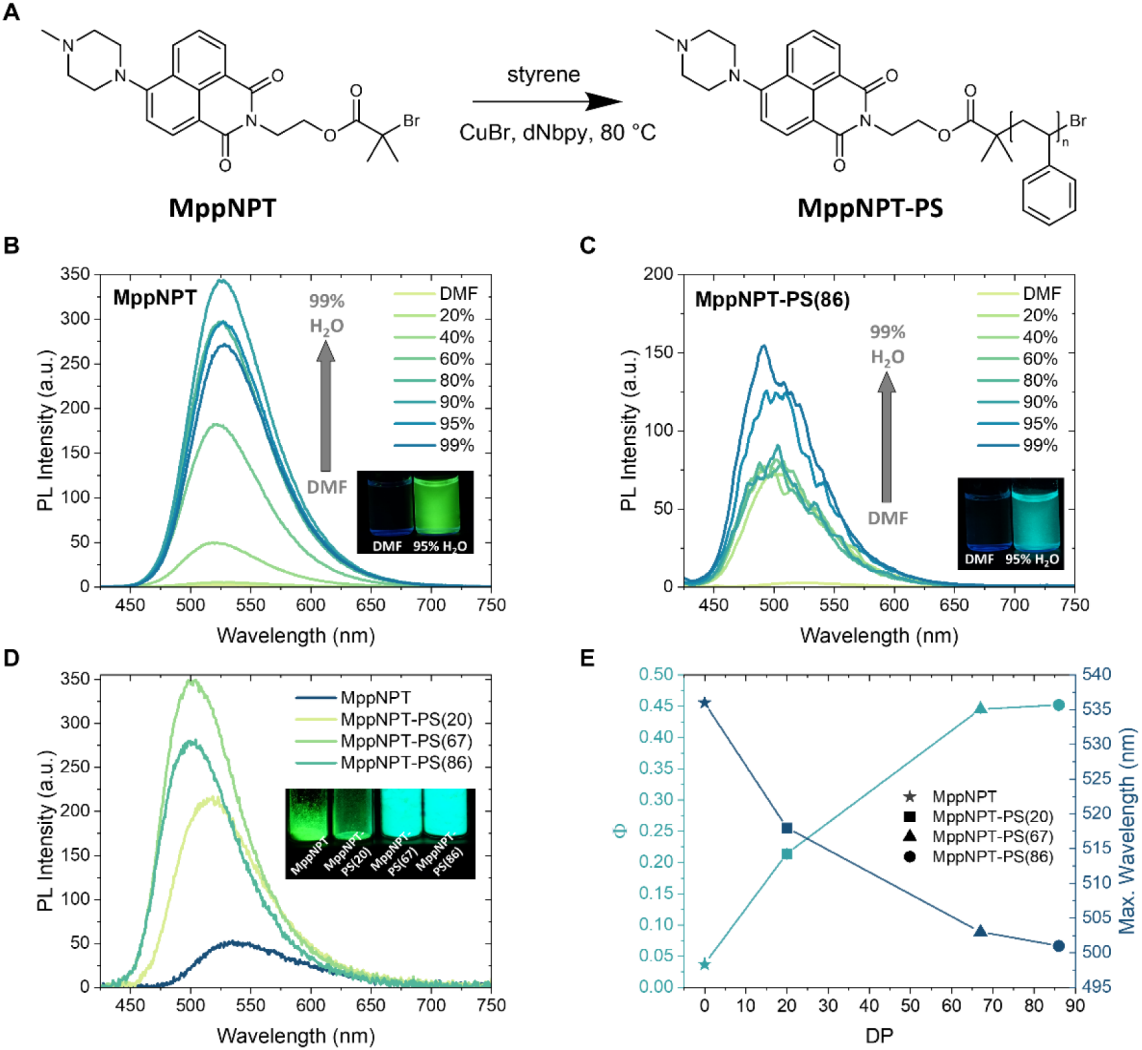
Photophysical properties of MppNPT and MppNPT-PS. (A) Chemical structure of the initiator MppNPT and the polymer MppNPT-PS via ATRP. (B) PL spectra of MppNPT in DMF and DMF/water mixtures with different water fractions. Inset: Photograph of MppNPT solutions under UV light (365 nm). (C) PL spectra of MppNPT-PS(86) in DMF and DMF/water mixtures with different water fractions. Inset: Photograph of MppNPT-PS(86) solutions under UV light (365 nm). The concentration was 5.0 μM. (D) PL spectra of solid films produced with MppNPT and MppNPT-PS of different DPs. Inset: Photograph of solid powders of MppNPT and MppNPT-PS under UV light (365 nm). (E) The solid-state Φ and wavelength of the maximum emission plotted against DP (DP = 0 corresponds to MppNPT). The excitation wavelength λ_ex_ = 400 nm.

After introducing polymer chains, MppNPT-PS with a DP of 86 displayed similar AIE behavior when raising the water fraction in DMF, along with a blue-shifted emission that was not observed in the initiator MppNPT (Figure 5C). This also applies to the DMF/D_2_O solutions (Figure S9). Although the polymers exhibited lower PL intensity compared to MppNPT, possibly due to precipitation or sedimentation of polymer aggregates, all of them showed enhanced emission in their aggregate state compared to the nonemissive MppNPT in the dissolved state (Figure S11). In addition, the polymers showed slightly blue-shifted absorption spectra (Figure S10), indicating the suppression of TICT states even with short polymer chains, e.g., MppNPT-PS with a DP of 20.

This has also been reflected in the drastic increase of solid-state Φ upon growing polymer chains with a DP of 20 (Φ = 0.21), compared to the rather low solid-state Φ of only 0.04 in MppNPT, likely due to the joint effect of TICT and PET within the scaffold (Figure 5D,E). Further chain extension to DP 86 provided higher Φ values up to 0.45, which is over 10-fold enhancement compared to the small molecule. Despite the increase, the Φ values are significantly lower than those of MphNPT polymers, likely due to fluorescence quenching in the PET pathway that is more sensitive to donor–acceptor distance.^41^ Concurrently, the emission maxima blue-shifted from 536 to 501 nm as DP increased, consistent with the nonpolar microenvironments imparted by polymer chains. TRPL measurements of the MppNPT initiator and polymer (Figure S12) also showed increased emission lifetimes in the aggregate state for both, with especially the amplitude of the fast LE component decreasing relative to the slower components with larger water fractions (Table S2). This indicates that both the initiator and polymer emitted via AIE. Together, these results indicate the crucial role of polymer chains in enhancing solid-state emission by effectively suppressing fluorescence quenching arising from TICT and PET processes.

Additionally, copolymers containing styrene and 1-vinylnaphthalene were also prepared, denoted as MppNPT-*co*Naph­(*x*/*y*), where *x*, *y* is the calculated DP of styrene and 1-vinylnaphthalene, respectively (Figure S13). These copolymers also exhibited enhanced emission in the aggregate state. Notably, MppNPT-*co*Naph­(19/1.1) showed higher solid-state Φ compared to MppNPT-PS(20) at similar dye concentration (Φ = 0.26 vs 0.21, Figure S14), and a blue-shifted emission maximum (511 vs 518 nm), indicating the additional steric hindrance contributed to AIE due to the larger aromatic units at minimal concentration. Altogether, these results demonstrate that the introduction of nonpolar, rigid polymer chains successfully light up the emission of TICT fluorophores even with additional PET quenching effect, indicating the potential of oligomerization- or polymerization-inhibited TICT in achieving strong molecular aggregate emission. Further work on applying this strategy to other TICT fluorophore systems beyond NPT is worth investigation.

## Conclusions

We introduced a simple and convenient approach to enhance the aggregate emission of TICT fluorophores by incorporating nonpolar, rigid polymer chains through single dye-initiated ATRP. Using naphthalimide-based TICT dyes as model fluorophores, we demonstrated that polymerization can effectively transform inherently ACQ fluorophores into AIE-active macromolecular systems. Notably, the fluorescence quantum yield in solid films reached an impressive 0.80, while maintaining MphNPT dye concentrations of nearly 20 wt %.

The incorporation of polystyrene chains plays a crucial role in offering steric hindrance that suppresses TICT, thereby significantly enhancing fluorescence in both aggregated and solid states. The emission wavelength exhibited a blue shift from 540 to 500 nm with increased fluorescence lifetimes, further indicating the impact of polymer chains in providing a nonpolar microenvironment that inhibits TICT processes. Additionally, when this strategy was applied to an AIE-active NPT system that initially exhibited only weak emission in the solid state, the resulting polymers, MppNPT-PS, achieved solid-state quantum yields of up to 0.45, despite the presence of strong PET-induced quenching effects.

Our strategy provides a simple and cost-effective approach for achieving strong molecular emission, circumventing the need for expensive or toxic reagents and complex synthetic processes typically associated with electron donor engineering. The findings highlight the potential of polymerization-inhibited TICT as a robust tool for developing highly fluorescent aggregate materials,^42–44^ with potential applications in optoelectronics such as luminescent solar concentrators^25,45^ and waveguiding devices.^23,46^

This work opens new avenues for exploring the applicability of polymerization strategies to a broader range of TICT fluorophore systems beyond naphthalimide derivatives, such as coumarin- or rhodamine-based dyes. The ability to achieve enhanced emission through structural modification and controlled polymerization represents an important direction in the design of fluorescent materials with tailored properties.

## Supplementary Material



## Notes

The detailed polymerization conditions and characterizations of initiators and polymers, including NMR and SEC spectra, are provided in Table S1 and Figures S15–S24. Additional photophysical characterizations of the initiators and polymers, including photoluminescence spectra, absorption spectra, and TRPL measurements, are provided in Tables S2 and S3 and Figures S1–S14 (PDF)
