# Supplementary material for: Polymerization-Inhibited Twisted Intramolecular Charge Transfer for Strong Molecular Aggregate Emission
Source: ACS Polym Au. 2025 Nov 5;6(2):565–74. doi: 10.1021/acspolymersau.5c00123 (PMC13067170; doi:10.1021/acspolymersau.5c00123)
Supplement: Supplementary file 1 [file lg5c00123_si_001.pdf]

Supporting Information for

Polymerization-inhibited Twisted Intramolecular Charge Transfer for Strong Molecular Aggregate Emission

Suiying Ye[1], Carolina Söll[1], Wanqing Cao[2], Benjamin Choiselat[2], Ramsha Khan,[3] Tero-Petri Ruoko,[3] Yinyin Bao[*, 1, 2]

[1]Department of Chemistry and Applied Biosciences, ETH Zurich, Vladimir-Prelog-Weg 1-5/10, 8093 Zurich, Switzerland.
[2]Department of Chemistry, University of Helsinki, A. I. Virtasen aukio 1, 00014 Helsinki, Finland
[3]Chemistry and Advanced Materials, Faculty of Engineering and Natural Sciences, Tampere University, 33720 Tampere, Finland

[*]Corresponding author. Email: ybao@ethz.ch; yinyin.bao@helsinki.fi

Table S1. Polymerization conditions and polymer characterizations.

Sample	[St]/[Naph]/[I]	Temperature (°C)	Time (h)	χ^a_{St}	χ^a_{Naph}	DP_{st}^b	DP_{Naph}^b	Naph%^{c}	$M_{n, NMR}^d$ (g.mol^{-1})	$M_{n, SEC}$ (g.mol^{-1})	$Đ$
**MphNPT-PS(12)**	80/0/1	80	2.5	14%	-^{e}	12	-^{e}	-^{e}	1730	2710	1.06
**MphNPT-PS(18)**	30/0/1	80	overnight	24%	-^{e}	18	-^{e}	-^{e}	2350	3210	1.05
**MphNPT-PS(20)**	80/0/1	80	5	23%	-^{e}	20	-^{e}	-^{e}	2560	3340	1.05
**MphNPT-PS(64)**	80/0/1	80	overnight	85%	-^{e}	64	-^{e}	-^{e}	7140	6520	1.04
**MphNPT-*co*Naph(14/1.1)**	95/5/1	80	overnight	26%	64%	14	1.1	7%	2100	2920	1.17
**MphNPT-*co*Naph(26/0.3)**	99/1/1	80	overnight	36%	67%	26	0.3	1%	3230	3830	1.05
**MppNPT-PS(20)**	80/0/1	80	5	19%	-^{e}	20	-^{e}	-^{e}	2570	3080	1.05
**MppNPT-PS(29)**	80/0/1	115	1	33%	-^{e}	29	-^{e}	-^{e}	3510	3730	1.04
**MppNPT-PS(67)**	120/0/1	80	overnight	43%	-^{e}	67	-^{e}	-^{e}	7470	5500	1.04
**MppNPT-PS(86)**	80/0/1	115	5	80%	-^{e}	86	-^{e}	-^{e}	9450	6950	1.07
**MppNPT-*co*Naph(19/1.2)**	95/5/1	80	overnight	22%	61%	19	1.2	6%	2650	2790	1.11
**MppNPT-*co*Naph(32/0.3)**	99/1/1	80	overnight	26%	71%	32	0.3	1%	3870	3910	1.05

Note: [a]Polymerization conversion (χ) was calculated from NMR spectra of the reaction mixture before purification, using $\chi = \frac{I_{polymer}}{I_{polymer}+I_{monomer}}$, *where the area under the peaks at 5.80,5.22 ppm (β-protons of vinyl monomers) was used to quantify the remained monomer amounts, and 6.50-7.50 ppm (phenyl protons) to quantify both monomer and polymer amounts. [b]Degree of polymerization (DP) was quantified from NMR spectra of purified polymer samples, using the area under the peaks at 6.50-7.50 ppm (phenyl protons of benzyl units), 7.50-8.00 ppm (phenyl protons of naphthalene units), and 8.54 ppm (phenyl protons of NPT). [c]The Naph content was calculated using* $Naph\% = \frac{DP_{Naph}}{DP_{St}+DP_{Naph}}$. [d]$M_{n, NMR} = DP_{St} \times 104.15 + DP_{Naph} \times 154.21 + 475.3$ *for MphNPT polymers or* $M_{n, NMR} = DP_{St} \times 104.15 + DP_{Naph} \times 154.21 + 488.4$ *for MppNPT polymers. [e]Not applicable.*

Table S2. Photophysical characterizations of the initiators and polymers containing naphthalimide.

Sample	λ_{film}[a](nm)	Φ_{film}	λ_{aggre}[b] (nm)	dye wt%[c]
**MphNPT**	533	43.6% ±0.00%	546	100%
**MphNPT-PS(12)**	510	50.5% ±0.10%	513	27.6%
**MphNPT-PS(18)**	-[d]	-[d]	510	20.2%
**MphNPT-PS(20)**	504	71.1% ±0.17%	509	18.6%
**MphNPT-PS(64)**	497	79.7% ±0.17%	512	6.7%
**MphNPT-*co*Naph(14/1.1)**	508	60.6% ±0.20%	508	22.6%
**MphNPT-*co*Naph(26/0.3)**	506	73.0% ±0.06%	504	14.7%
**MppNPT**	536	3.7% ±0.06%	527	100%
**MppNPT-PS(20)**	518	21.4% ±0.06%	509	19.0%
**MppNPT-PS(29)**	-[d]	-[d]	500	13.9%
**MppNPT-PS(67)**	503	44.5% ±0.12%	497	6.5%
**MppNPT-PS(86)**	501	45.2% ±0.06%	493	5.2%
**MppNPT-*co*Naph(19/1.2)**	511	26.2% ±0.06%	505	18.4%
**MppNPT-*co*Naph(32/0.3)**	505	34.5% ±0.12%	500	12.6%

Note: [a]The emission wavelength at a maximum PL intensity from thin film samples. [b]The emission wavelength at a maximum PL intensity from samples in DMF/water (5/95, v/v). [c]The dye content in weight fraction (wt. %) was calculated as $M_{initiator}/M_{n,NMR,polymer}$. *[d]Not measured due to the similar DP.*

Table S3. TRPL lifetimes of the initiators and the polymers in DMF and DMF/water mixtures with different water fractions. The data were fit to either a two- (initiators) or three-exponential (polymers) decay model with instrument response function reconvolution: $I(t) = \sum_{i=1}^{n} A_i e^{-t/\tau_i}$. The intensity-weighted average lifetime was determined as $\tau_{avg} = \sum_{i=1}^{n} \frac{A_i \tau_i^2}{A_i \tau_i}$.

Sample	DMF/water (v/v)	A_1 rel.	A_2 rel.	A_3 rel.	τ_1 (ns)	τ_2 (ns)	τ_3 (ns)	τ_{avg} (ns)	χ^2
**MphNPT**	100/0	98.8	1.3	-[a]	0.881±0.002	7.7±0.3	-[a]	1.57±0.07	1.464
	80/20	94.8	5.3	-[a]	0.357±0.003	5.49±0.02	-[a]	2.726±0.008	1.975
	60/40	98.8	1.3	-[a]	0.235±0.001	6.62±0.03	-[a]	1.932±0.009	1.657
	40/60	99.0	1.1	-[a]	0.156±0.001	6.61±0.03	-[a]	2.110±0.007	1.859
	20/80	97.7	2.4	-[a]	0.144±0.001	6.38±0.03	-[a]	3.38±0.01	2.523
	5/95	96.7	3.4	-[a]	0.138±0.002	4.29±0.03	-[a]	2.28±0.01	9.145
	1/99	94.7	5.4	-[a]	0.183±0.003	4.39±0.02	-[a]	2.61±0.01	5.412
**MphNPT-PS(18)**	100/0	32.7	66.4	1.1	0.64±0.01	1.596±0.006	7.65±0.09	1.810±0.002	1.281
	80/20	38.2	32.9	29.1	0.72±0.02	4.54±0.11	9.32±0.06	7.198±0.006	1.296
	60/40	36.4	35.3	28.4	0.66±0.02	4.3±0.2	10.01±0.07	7.638±0.009	1.334
	40/60	35.5	37.2	27.5	0.76±0.03	4.43±0.08	10.27±0.05	7.701±0.005	1.313
	20/80	38.9	35.5	25.7	0.73±0.03	4.3±0.2	10.2±0.1	7.585±0.008	1.228
	5/95	37.7	36.8	25.6	0.83±0.04	4.6±0.2	10.57±0.09	7.775±0.005	1.224
	1/99	41.0	33.3	25.9	0.63±0.04	4.2±.02	10.25±0.09	7.706±0.005	1.274
**MppNPT**	100/0	96.2	3.9	-[a]	0.112±0.004	6.91±0.02	-[a]	4.97±0.02	3.950
	80/20	85.9	14.2	-[a]	0.108±0.003	8.086±0.008	-[a]	7.487±0.007	1.825
	60/40	24	77	-[a]	0.30±1.9	8.2±0.1	-[a]	8.08±0.03	1.484
	40/60	21.0	79.1	-[a]	0.48±0.04	8.018±0.009	-[a]	7.901±0.007	1.324
	20/80	18.5	81.6	-[a]	0.73±0.03	7.806±0.008	-[a]	7.658±0.005	1.216
	5/95	17.5	82.6	-[a]	0.8±0.1	7.49±0.02	-[a]	7.346±0.006	1.210
	1/99	13.2	86.9	-[a]	1.3±0.3	7.36±0.02	-[a]	7.198±0.008	1.300
**MppNPT-PS(67)**	100/0	93.4	3.9	2.8	0.078±0.001	2.07±0.02	8.79±0.002	5.81±0.01	1.390
	80/20	48.6	37.6	13.9	0.56±0.02	2.86±0.03	7.39±0.03	4.549±0.005	1.309
	60/40	44.3	38.4	17.4	0.52±0.01	2.98±0.05	7.10±0.06	4.714±0.004	1.395
	40/60	40.4	37.2	22.5	0.54±0.01	3.28±0.06	7.69±0.05	5.492±0.005	1.164
	20/80	43.8	36.8	19.5	0.55±0.02	3.44±0.08	7.86±0.10	5.443±0.005	1.303
	5/95	39.5	37.4	23.7	0.56±0.02	3.55±0.06	8.35±0.05	6.069±0.008	1.243
	1/99	38.6	40.0	22.7	0.61±0.02	3.53±0.07	8.05±0.05	5.736±0.007	1.368

[a]*A two-exponential decay model was applied, therefore, A_3 and τ_3 are not applicable.*

Supplementary Figures

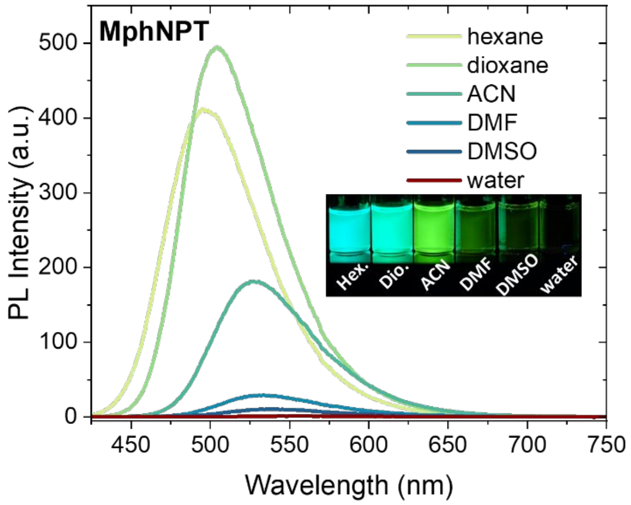

Figure S1. Photoluminescence (PL) spectra of MphNPT in different solvents. Inset: Photograph of MphNPT in different solvents under UV light (365 nm). The concentration was 5.0 μM. The excitation wavelength λ_{ex} = 400 nm.

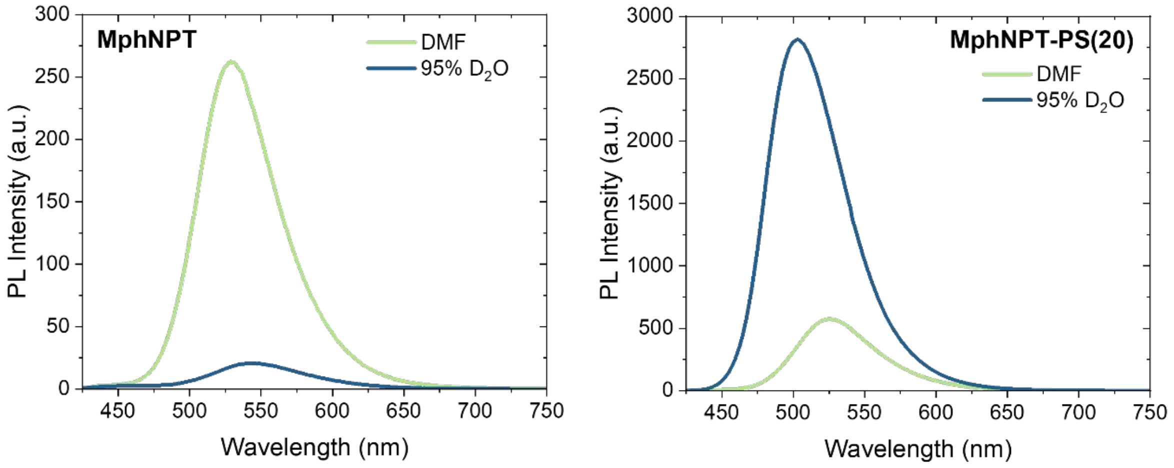

Figure S2. PL spectra of MphNPT and MphNPT-PS(20) in DMF or DMF/D_2O (5/95, v/v). The concentration was 10.0 μM. The excitation wavelength λ_{ex} = 400 nm.

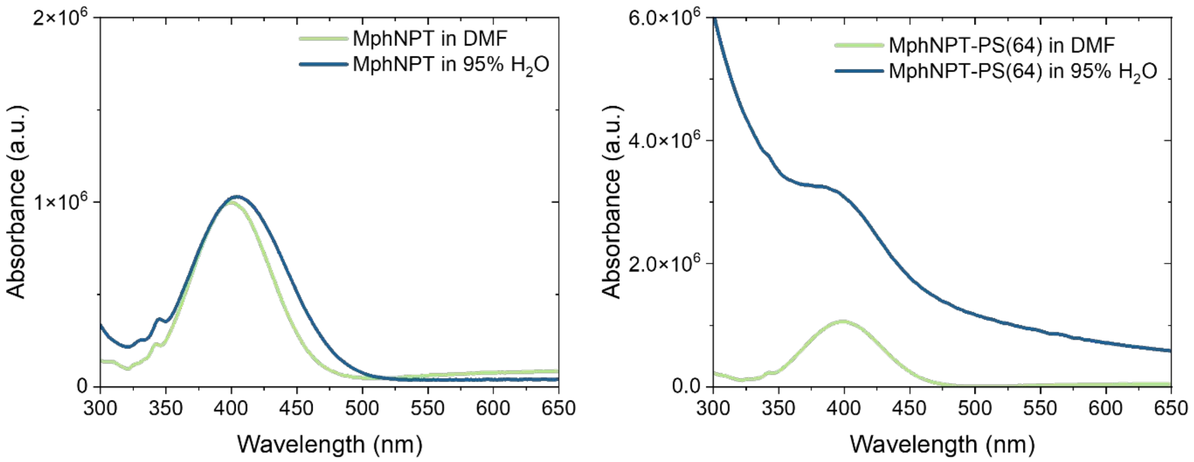

Figure S3. Absorption spectra of MphNPT (left) and MphNPT-PS(64) (right) in DMF or DMF/water (5/95, v/v). The concentration was 10.0 μM.

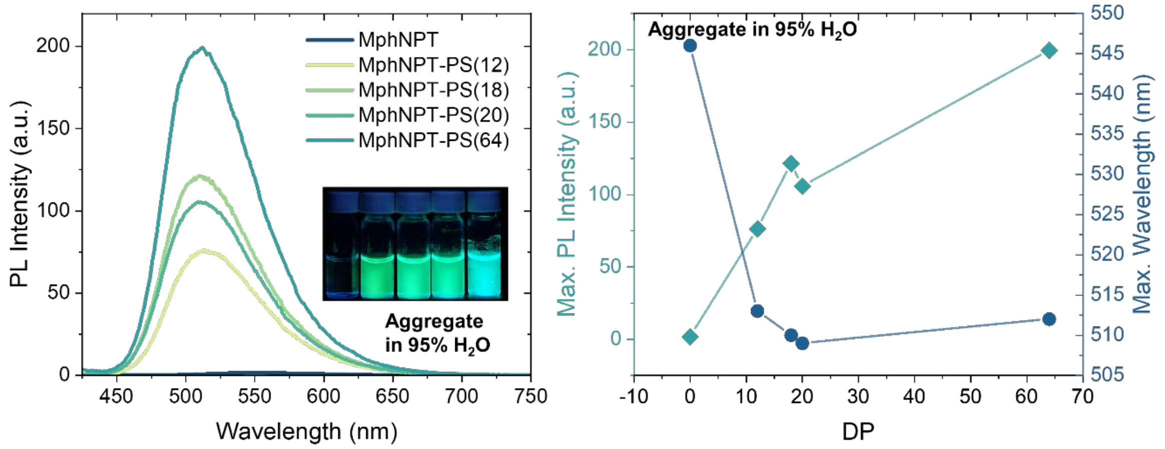

Figure S4. PL spectra (left) and comparison of maximum PL intensity and maximum emission wavelengths of MphNPT and MphNPT-PS of different DPs in DMF/water (5/95, v/v). Inset: Photograph of MphNPT and MphNPT-PS of different DPs in DMF/water (5/95, v/v) under UV light (365 nm). The concentration was 5.0 µM. The excitation wavelength λ_{ex} = 400 nm.

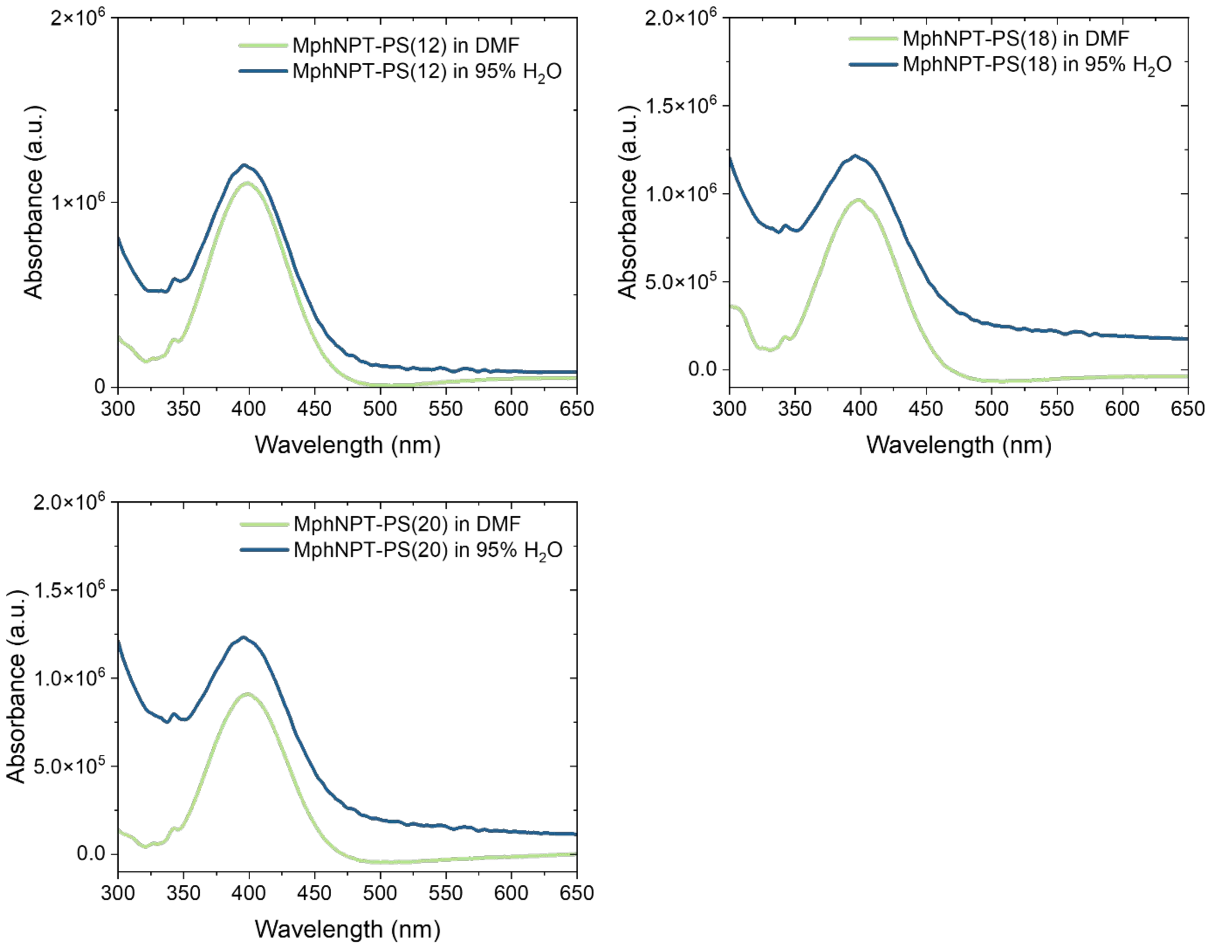

Figure S5. Absorption spectra of MphNPT-PS of different DPs in DMF or DMF/water (5/95, v/v). The concentration was 10.0 μM.

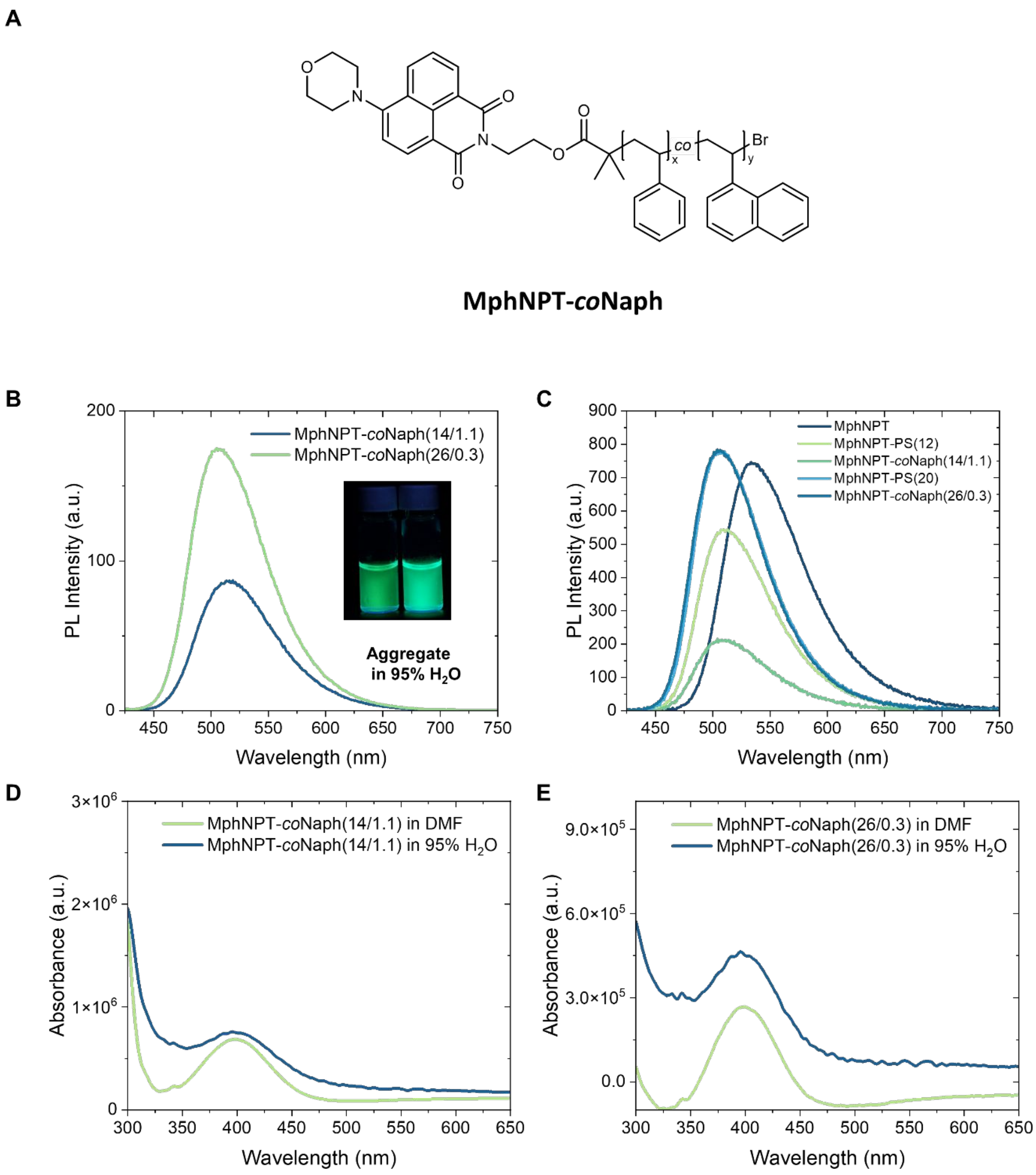

Figure S6. (A) Chemical structure of MphNPT-*co*Naph. (B) PL spectra MphNPT-*co*Naph in DMF/water (5/95, v/v). Inset: Photograph of MphNPT-*co*Naph in DMF/water (5/95, v/v) under UV light (365 nm). (C) PL spectra of solid films produced with MphNPT, MphNPT-PS, MphNPT-*co*Naph. (D and E) Absorption spectra of MphNPT-*co*Naph of different DPs in DMF or DMF/water (5/95, v/v). The concentration was 5.0 µM. The excitation wavelength λ_{ex} = 400 nm.

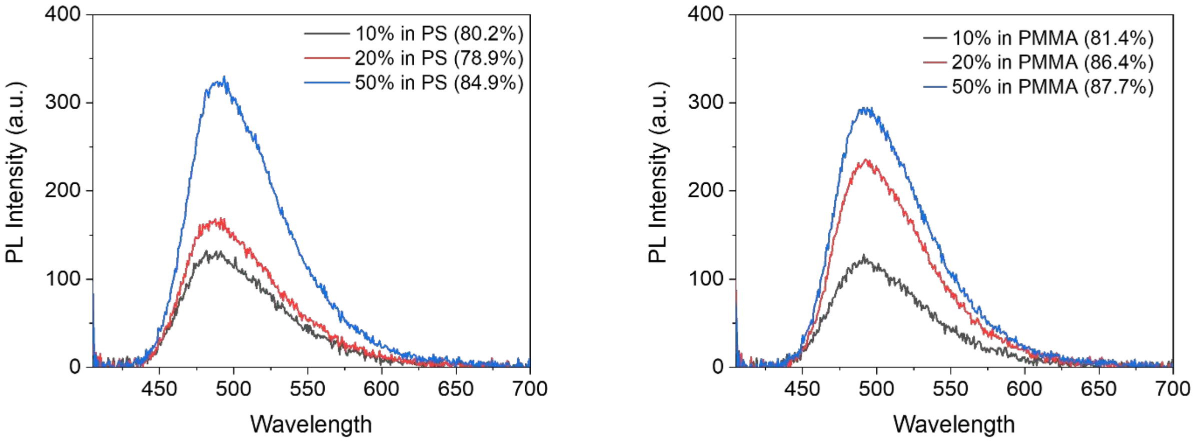

Figure S7. PL spectra of solid films produced with MphNPT-PS(64) mixed with polystyrene (left) or polymethyl methacrylate (right) at different wt.% of MphNPT-PS(64). The values in brackets indicate the corresponding PL quantum yield, Φ. The excitation wavelength λ_{ex} = 400 nm.

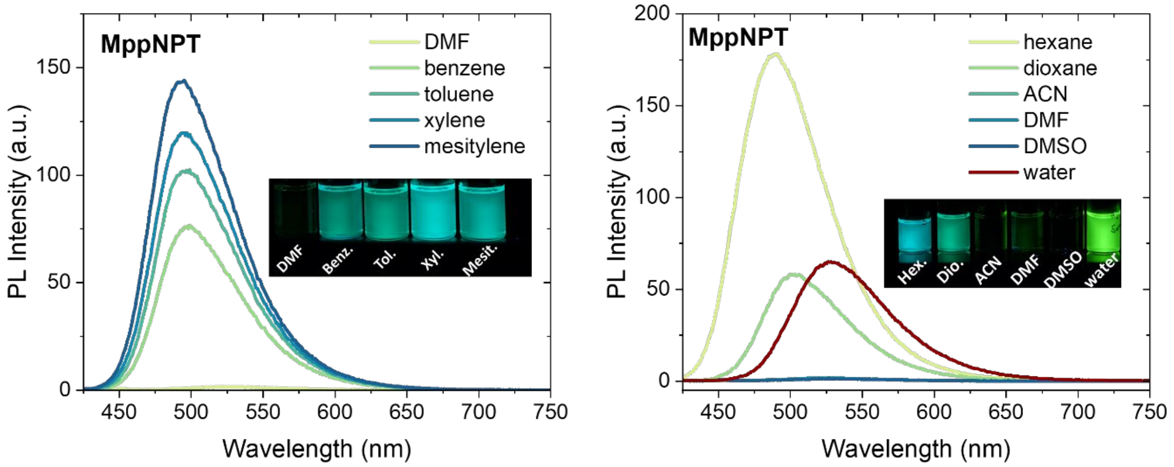

Figure S8. PL spectra of MppNPT in different solvents. Inset: Photograph of MppNPT in different solvents under UV light (365 nm). The concentration was 5.0 μM. The excitation wavelength λ_{ex} = 400 nm.

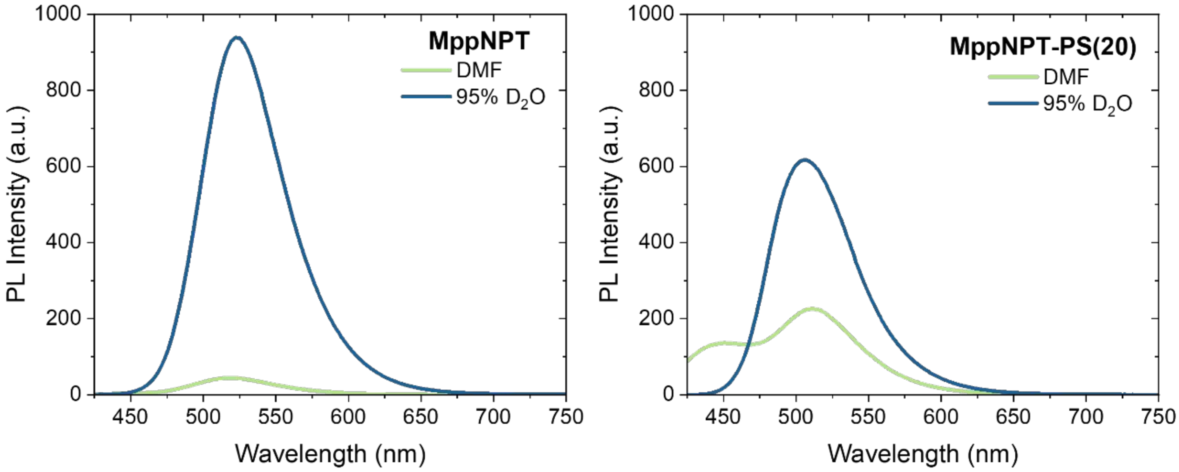

Figure S9. PL spectra of MppNPT and MppNPT-PS(20) in DMF or DMF/D_2O (5/95, v/v). The concentration was 10.0 μM. The excitation wavelength λ_{ex} = 400 nm.

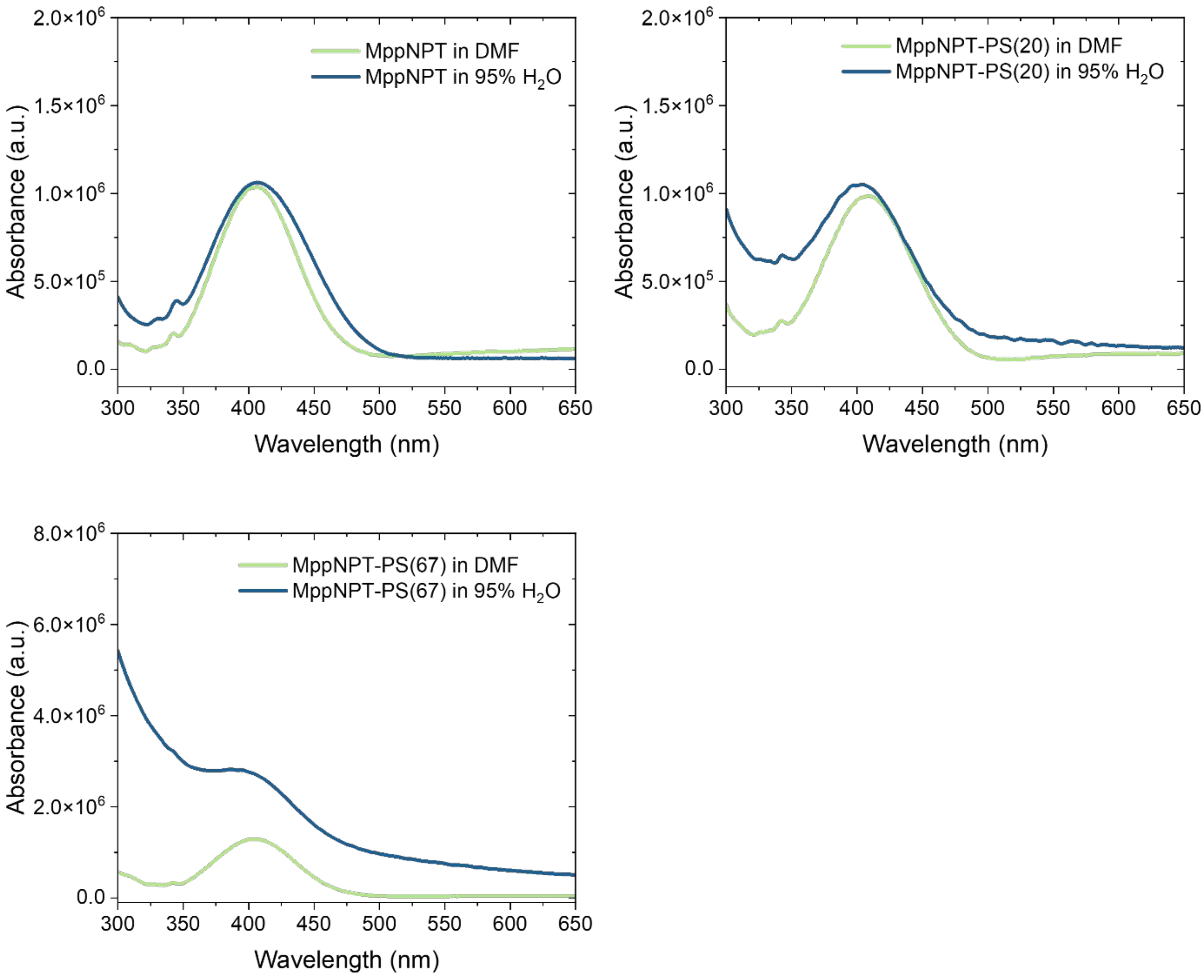

Figure S10. Absorption spectra of MppNPT and MppNPT-PS of different DPs in DMF or DMF/water (5/95, v/v). The concentration was 10.0 μM.

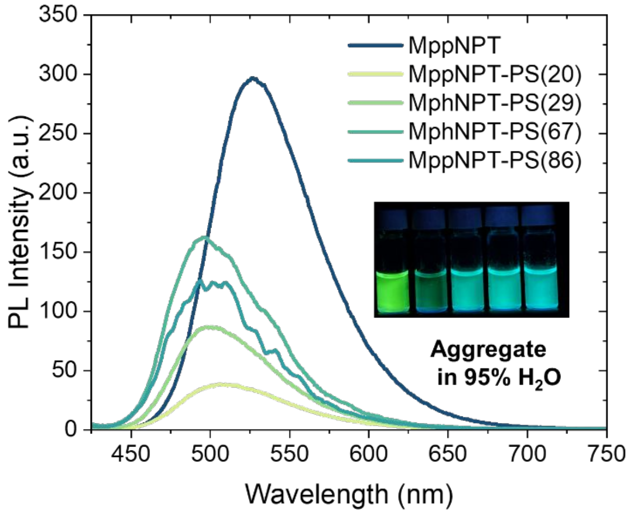

Figure S11. PL spectra of MppNPT and MppNPT-PS of different DPs in DMF/water (5/95, v/v). Inset: Photograph of MppNPT and MppNPT-PS of different DPs in DMF/water (5/95, v/v) under UV light (365 nm). The concentration was 5.0 μM. The excitation wavelength λ_{ex} = 400 nm.

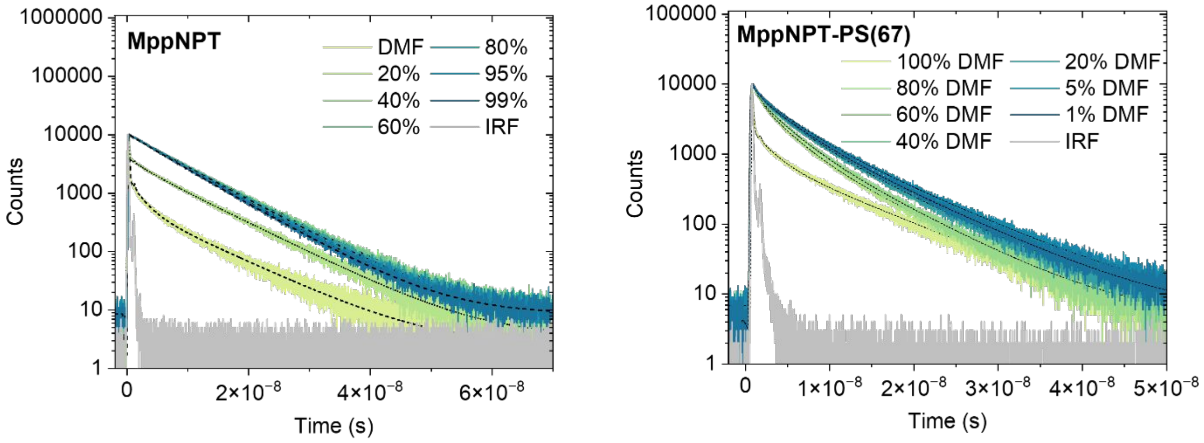

Figure S12. TRPL measurements of MppNPT and MppNPT-PS(67) in DMF and DMF/water mixtures with different water fractions. The concentration was 5.0 μM, the excitation wavelength was λ_{ex} = 401 nm, and the emission was monitored at 500 nm.

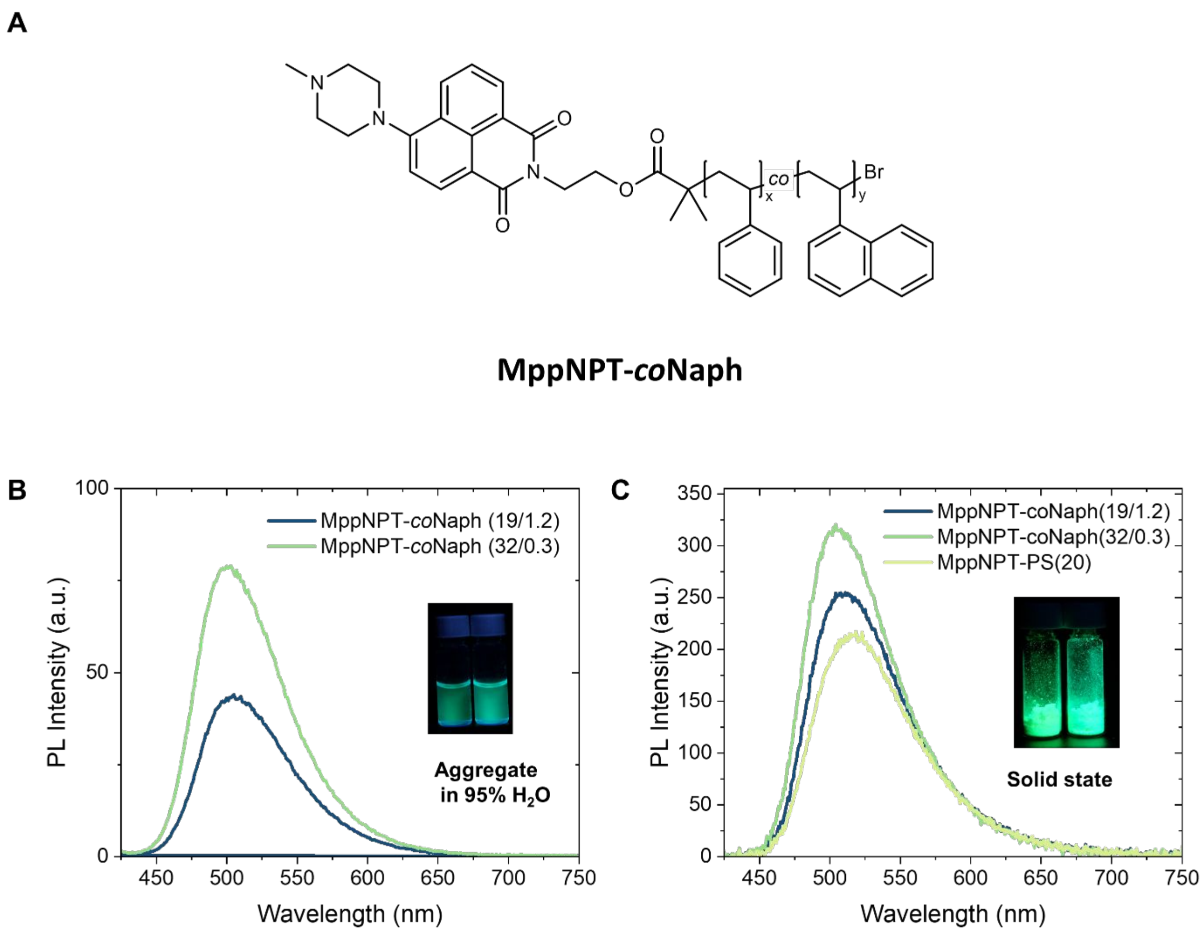

Figure S13. (A) Chemical structure of MppNPT-*co*Naph. (B) PL spectra MppNPT-*co*Naph in DMF/water (5/95, v/v). Inset: Photograph of MppNPT-*co*Naph in DMF/water (5/95, v/v) under UV light (365 nm). (C) PL spectra of solid films produced with MppNPT-PS, MppNPT-*co*Naph. Inset: Photograph of solid powders of MppNPT-*co*Naph under UV light (365 nm). The concentration was 5.0 µM. The excitation wavelength λ_{ex} = 400 nm.

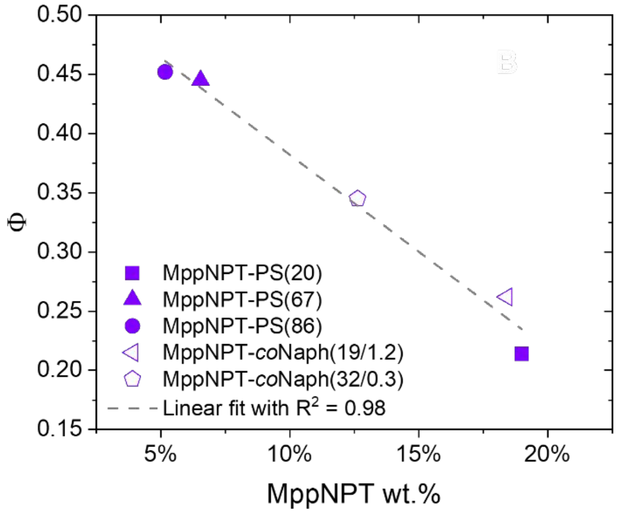

Figure S14. The solid-state PLQY, Φ, measured in solid films produced with MppNPT-PS and MppNPT-*co*Naph of different DPs, plotted against the weight fraction (wt.%) of MppNPT.

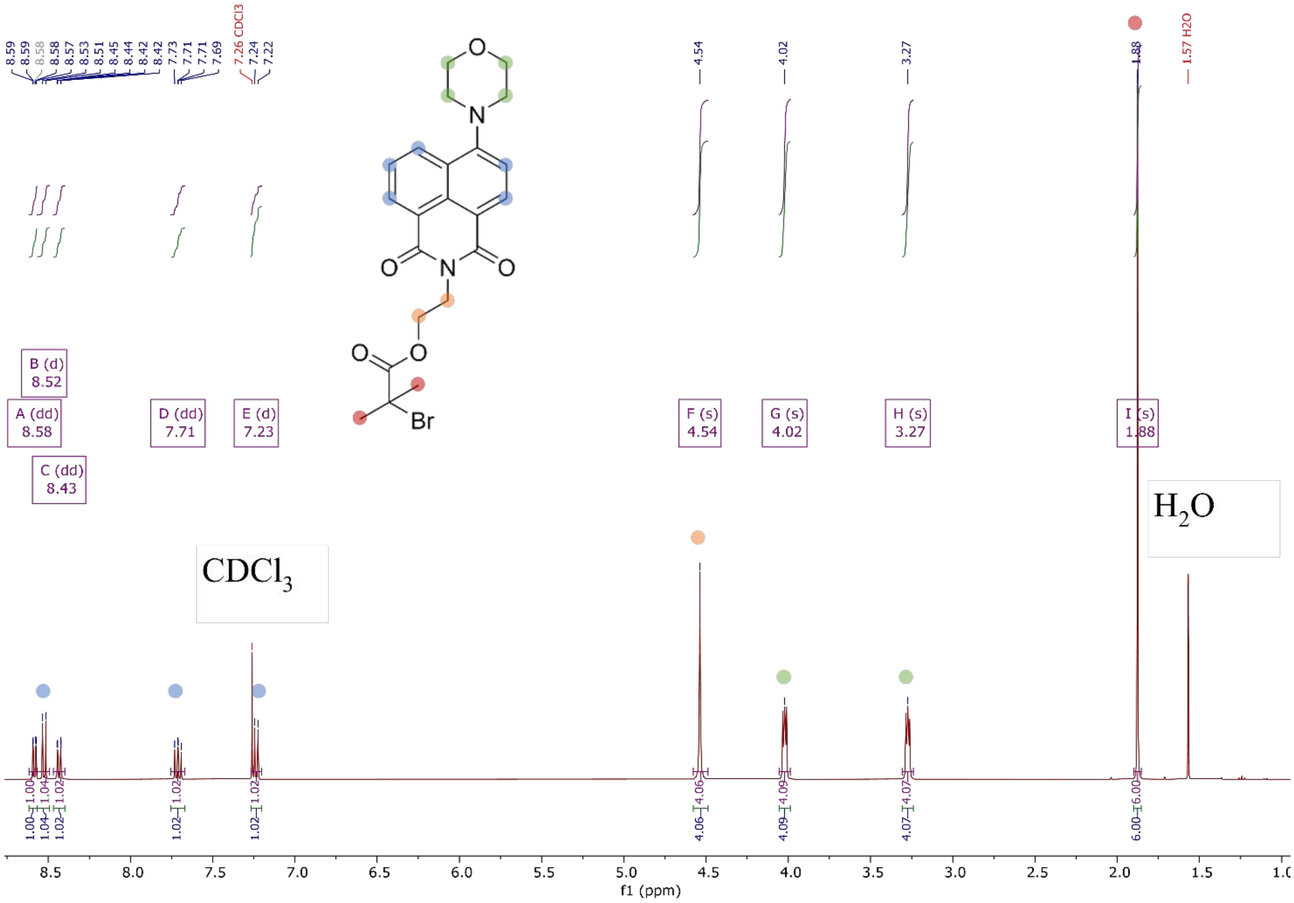

Figure S15. ^{1}H NMR spectrum of MphNPT (400 MHz, $CDCl_3$) δ 8.58 (dd, *J* = 7.3, 1.2 Hz, 1H), 8.52 (d, *J* = 8.1 Hz, 1H), 8.43 (dd, *J* = 8.5, 1.2 Hz, 1H), 7.71 (dd, *J* = 8.5, 7.3 Hz, 1H), 7.23 (d, *J* = 8.1 Hz, 1H), 4.54 (d, *J* = 2.0 Hz, 4H), 4.06 – 3.99 (m, 4H), 3.31 – 3.24 (m, 4H), 1.88 (s, 6H).

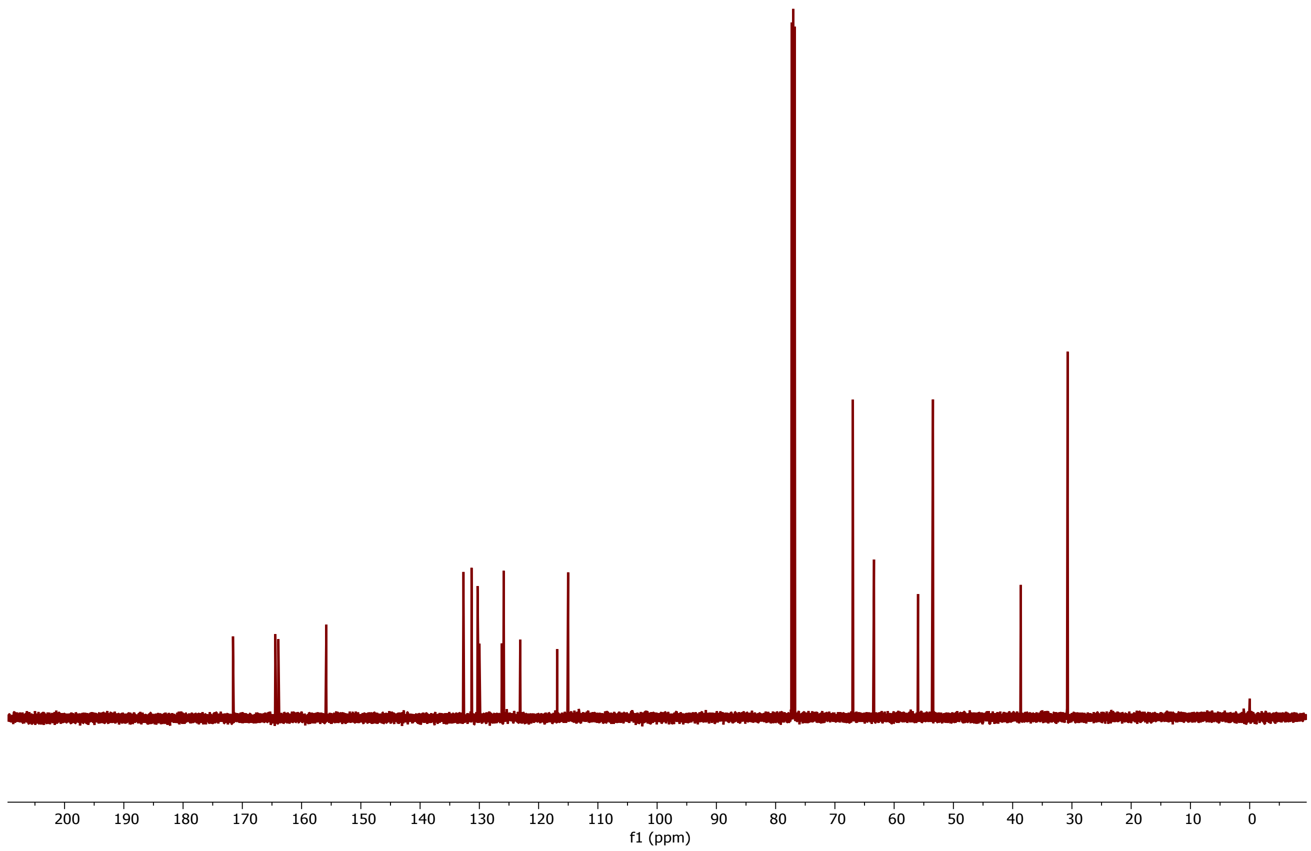

Figure S16. ^{13}C NMR spectrum of MphNPT (400 MHz, $CDCl_3$) δ = 171.6, 164.4, 163.9, 155.8, 132.7, 131.3, 130.3, 130.0, 126.2, 125.9, 123.1, 116.9, 115.0, 67.0, 63.4, 56.0, 53.5, 38.6, 30.7.

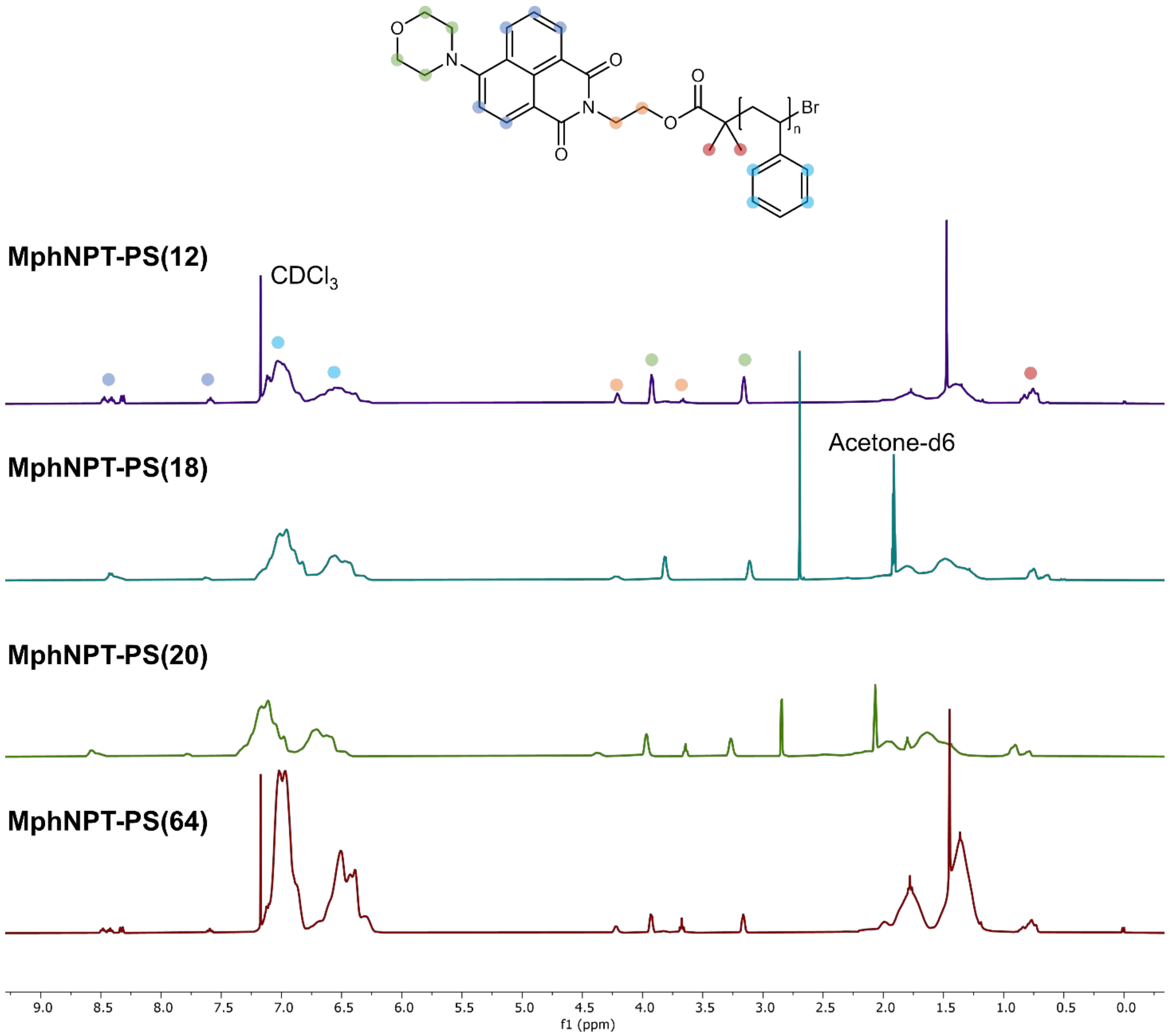

Figure S17. ^{1}H NMR spectra of MphNPT-PS of different DPs in either $CDCl_3$ or acetone-d6.

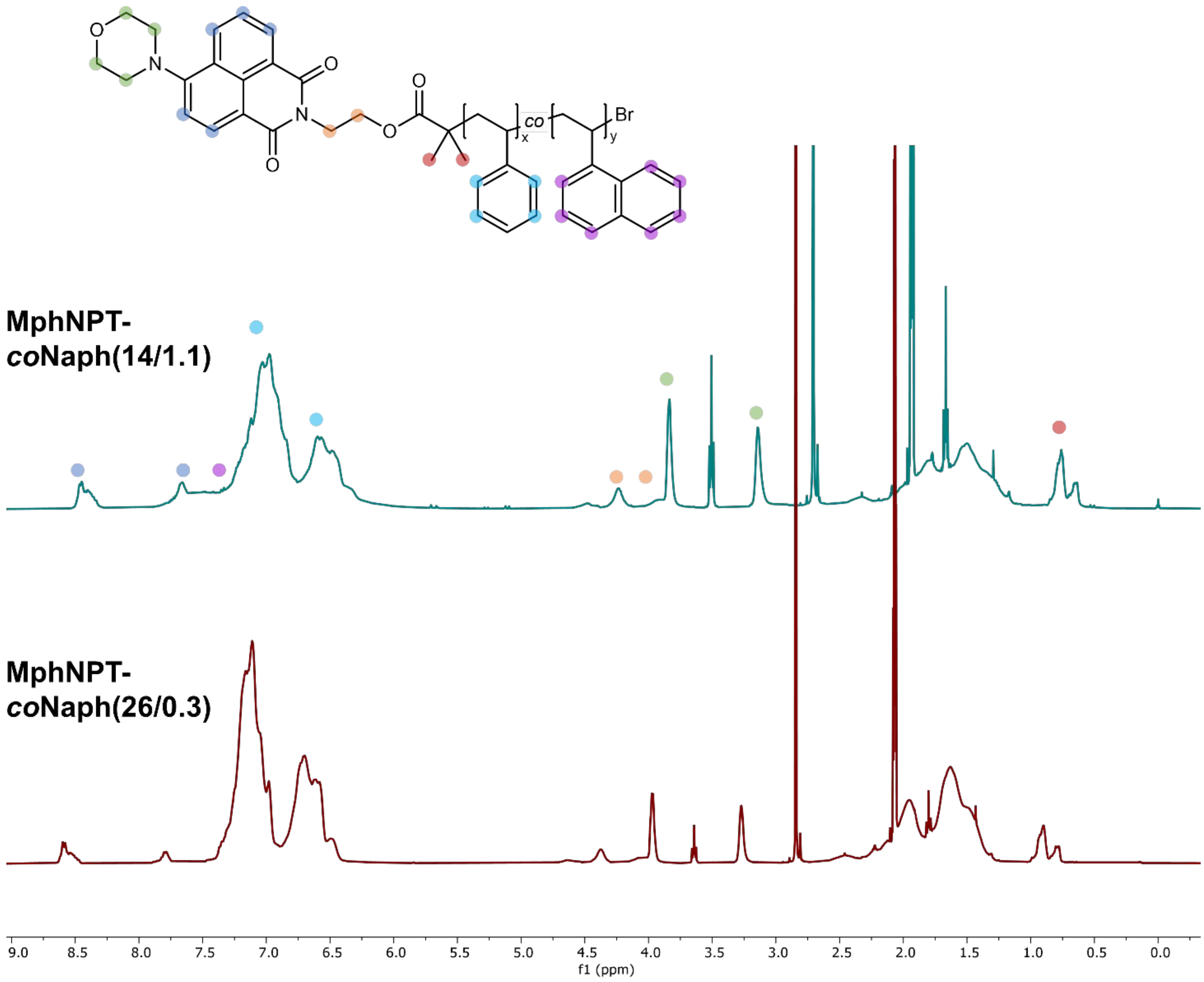

Figure S18. ^{1}H NMR spectra of MphNPT-*co*Naph of different DPs in acetone-d6.

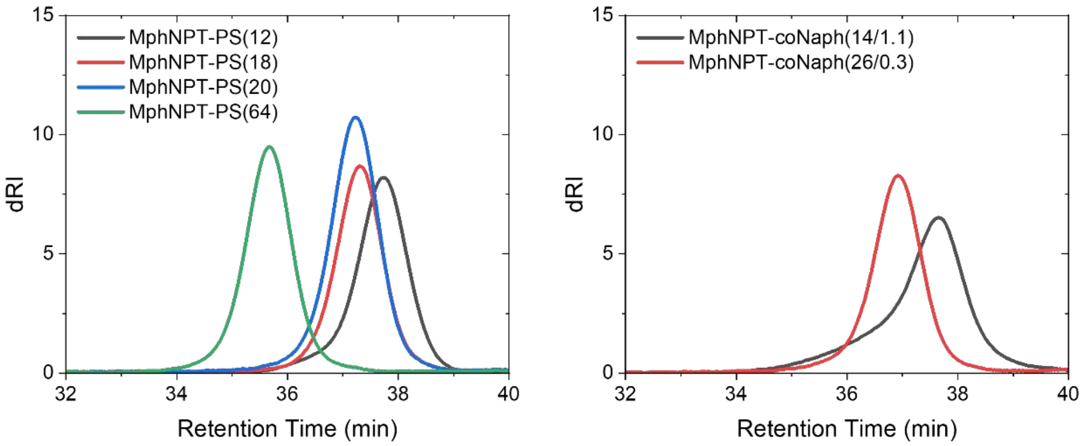

Figure S19. SEC traces from the differential refractive index (dRI) signal of MphNPT-PS and MphNPT-*co*Naph of different DPs.

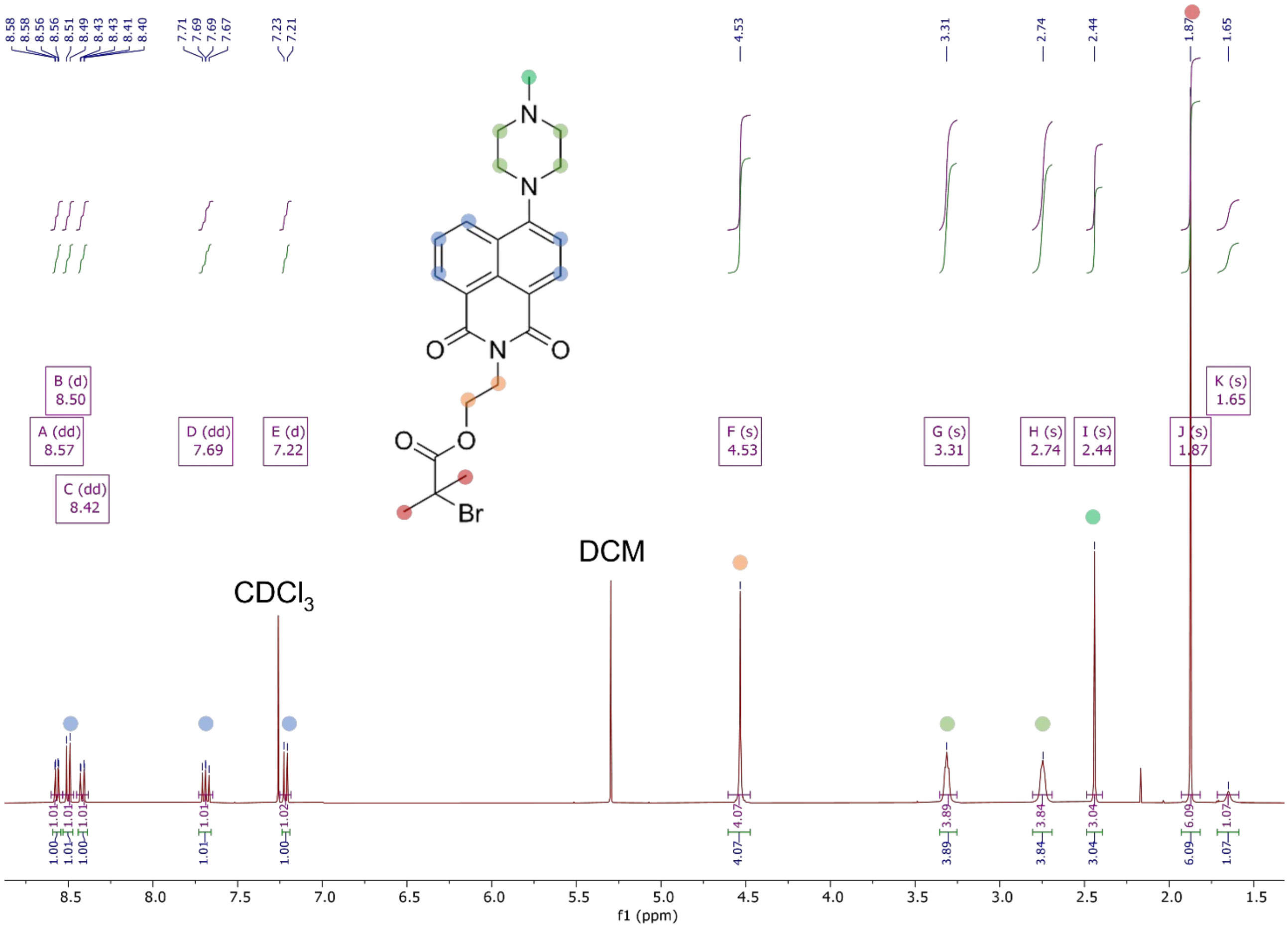

Figure S20. ^{1}H NMR spectrum of MppNPT (400 MHz, $CDCl_3$) δ 8.57 (dd, *J* = 7.3, 1.2 Hz, 1H), 8.50 (d, *J* = 8.1 Hz, 1H), 8.42 (dd, *J* = 8.5, 1.2 Hz, 1H), 7.69 (dd, *J* = 8.4, 7.3 Hz, 1H), 7.22 (d, *J* = 8.1 Hz, 1H), 4.54 (d, *J* = 1.9 Hz, 4H), 3.31 (t, *J* = 4.8 Hz, 4H), 2.75 (d, *J* = 5.2 Hz, 4H), 2.44 (s, 3H), 1.87 (s, 6H).

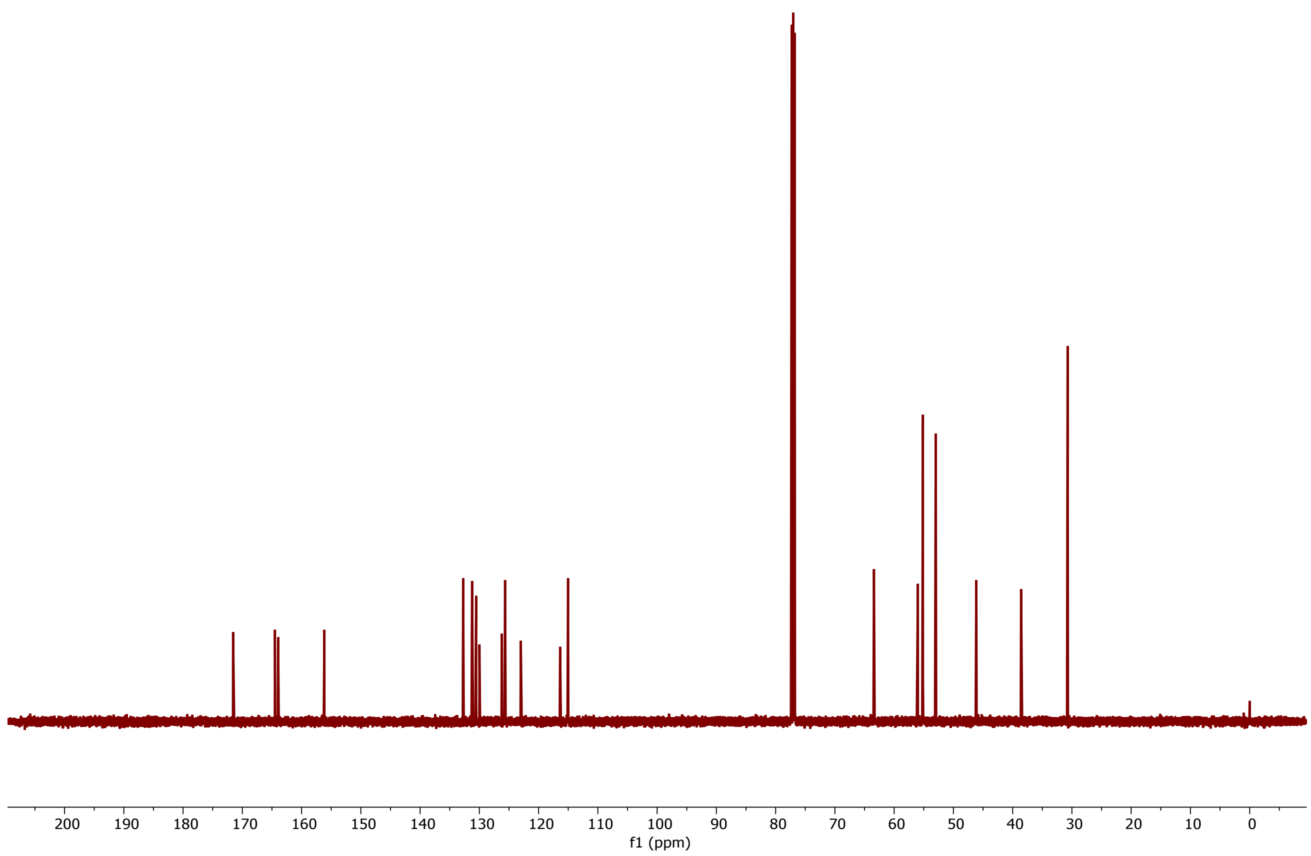

Figure S21. ^{13}C NMR spectrum of MppNPT (400 MHz, $CDCl_3$) δ = 171.6, 164.5, 164.0, 156.2, 132.7, 131.2, 130.6, 130.0, 126.2, 125.7, 123.0, 116.4, 115.0, 63.4, 56.0, 55.1, 53.0, 46.1, 38.6, 30.7.

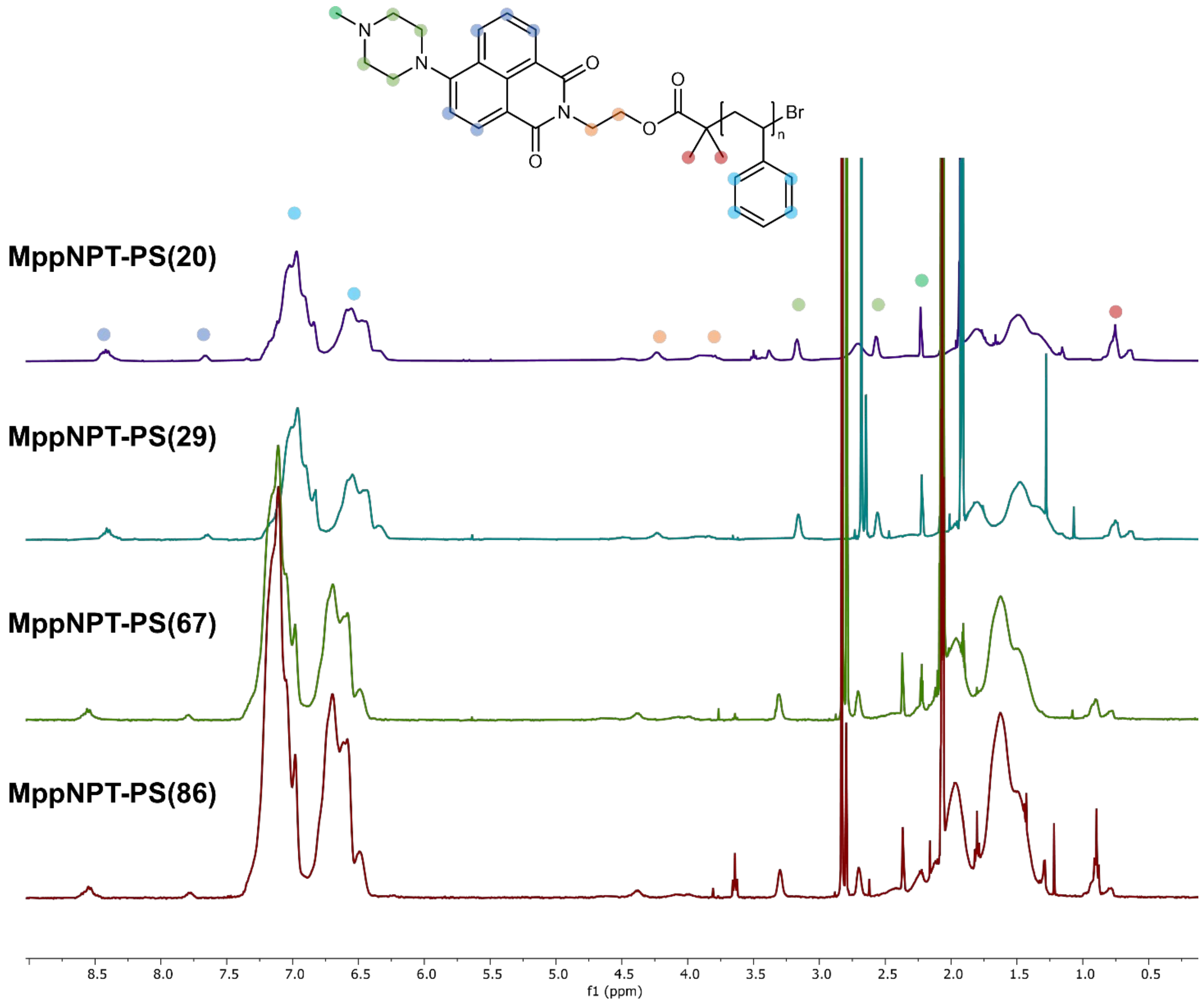

Figure S22. ^{1}H NMR spectra of MppNPT-PS of different DPs in acetone-d6.

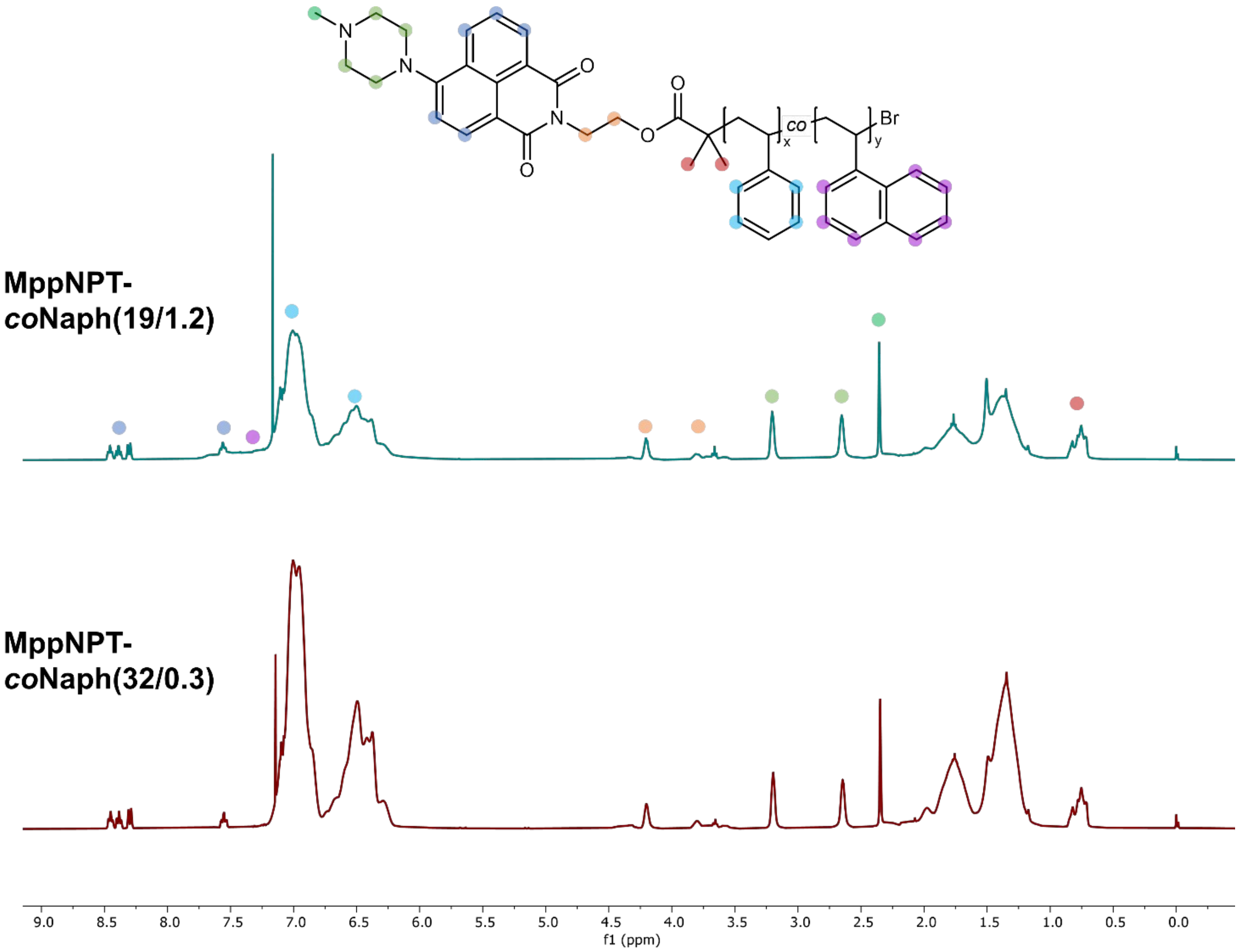

Figure S23. ^{1}H NMR spectra of MppNPT-*co*Naph of different DPs in $CDCl_3$.

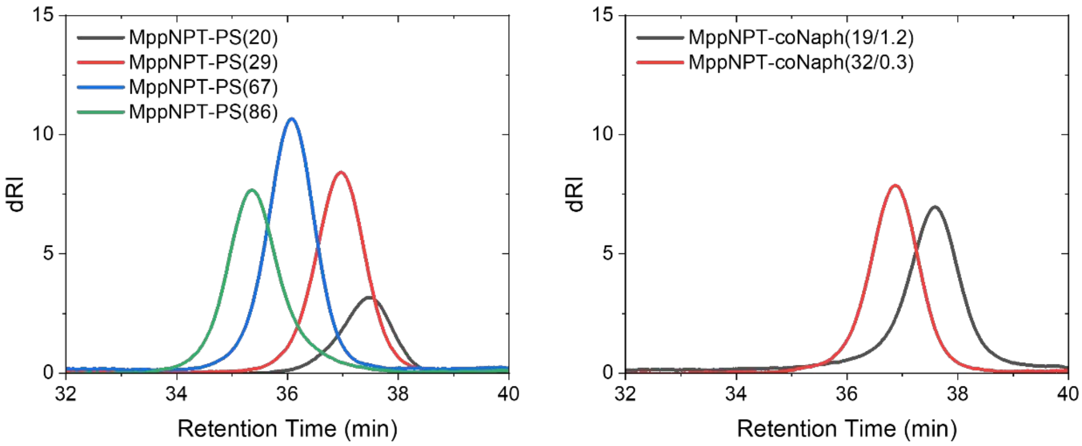

Figure S24. SEC traces from the differential refractive index (dRI) signal of MppNPT-PS and MppNPT-*co*Naph of different DPs.
